# Supplementary material for: Genetic Modifiers Associated with Vaso-Occlusive Crises and Acute Pain Phenomena in Sickle Cell Disease: A Scoping Review
Source: Int J Mol Sci. 2025 May 7;26(9):4456. doi: 10.3390/ijms26094456 (PMC12072568; doi:10.3390/ijms26094456)
Supplement: Supplementary file 1 [file ijms-26-04456-s001.zip › Supplementary Online Materials S1.pdf]

JBI CRITICAL APPRAISAL CHECKLIST FOR COHORT STUDIES

Reviewer: Froso Sophocleous Date: 2023-11-24

Author: Medeiros et al. (doi: 10.1590/1678-4685-GMB-2016-0161) Year: 2017 Record Number: AA

	Yes	No	Unclear	Not applicable
1. Were the groups similar and recruited from the same population?	X			
2. Were the exposures measured similarly to assign people to both exposed and unexposed groups?	X			
3. Was the exposure measured in a valid and reliable way?	X			
4. Were confounding factors identified?			X	
5. Were strategies to deal with confounding factors stated?			X	
6. Were the groups/participants free of the outcome at the start of the study (or at the moment of exposure)?			X	
7. Were the outcomes measured in a valid and reliable way?	X			
8. Was the follow up time reported and sufficient to be long enough for outcomes to occur?				X
9. Was follow up complete, and if not, were the reasons to loss to follow up described and explored?				X
10. Were strategies to address incomplete follow up utilized?				X
11. Was appropriate statistical analysis used?	X			

Overall appraisal: Include ☒ Exclude ☐ Seek further info ☐

Comments (Including reason for exclusion)

- The number of subjects included in the study and distributed within the groups is unclear
- Table 1 is not sufficiently described

Critical Appraisal Checklist for Cohort Studies - © JBI, 2020. All rights reserved. JBI grants use of these tools for research purposes only. All other enquiries should be sent to jbisynthesis@adelaide.edu.au.

JBI CRITICAL APPRAISAL CHECKLIST FOR COHORT STUDIES

Reviewer: __Froso Sophocleous__________________ Date: _2023-11-30 ___________________________

Author: Lakkakula et al. (DOI: 10.34172/npj.2022.10437) __Year: _2021 Record Number: AB

	Yes	No	Unclear	Not applicable
1. Were the groups similar and recruited from the same population?	☒	☐	☐	☐
2. Were the exposures measured similarly to assign people to both exposed and unexposed groups?	☒	☐	☐	☐
3. Was the exposure measured in a valid and reliable way?	☒	☐	☐	☐
4. Were confounding factors identified?	☒	☐	☐	☐
5. Were strategies to deal with confounding factors stated?	☒	☐	☐	☐
6. Were the groups/participants free of the outcome at the start of the study (or at the moment of exposure)?	☐	☒	☐	☐
7. Were the outcomes measured in a valid and reliable way?	☒	☐	☐	☐
8. Was the follow up time reported and sufficient to be long enough for outcomes to occur?	☐	☐	☐	☒
9. Was follow up complete, and if not, were the reasons to loss to follow up described and explored?	☐	☐	☐	☒
10. Were strategies to address incomplete follow up utilized?	☐	☐	☐	☒
11. Was appropriate statistical analysis used?	☒	☐	☐	☐

Overall appraisal: Include ☒ Exclude ☐ Seek further info ☐

Comments (Including reason for exclusion)

- n/a

Critical Appraisal Checklist for Cohort Studies - © JBI, 2020. All rights reserved. JBI grants use of these tools for research purposes only. All other enquiries should be sent to jbisynthesis@adelaide.edu.au.

JBI CRITICAL APPRAISAL CHECKLIST FOR COHORT STUDIES

Reviewer: Froso Sophocleous Date: 2023-11-30

Author: Figueiredo et al. (doi: 10.1371/journal.pone.0246067) Year: 2021 Record Number: AC

	Yes	No	Unclear	Not applicable
1. Were the groups similar and recruited from the same population?	☒	☐	☐	☐
2. Were the exposures measured similarly to assign people to both exposed and unexposed groups?	☒	☐	☐	☐
3. Was the exposure measured in a valid and reliable way?	☒	☐	☐	☐
4. Were confounding factors identified?	☒	☐	☐	☐
5. Were strategies to deal with confounding factors stated?	☒	☐	☐	☐
6. Were the groups/participants free of the outcome at the start of the study (or at the moment of exposure)?	☐	☒	☐	☐
7. Were the outcomes measured in a valid and reliable way?	☒	☐	☐	☐
8. Was the follow up time reported and sufficient to be long enough for outcomes to occur?	☐	☐	☐	☒
9. Was follow up complete, and if not, were the reasons to loss to follow up described and explored?	☐	☐	☐	☒
10. Were strategies to address incomplete follow up utilized?	☐	☐	☐	☒
11. Was appropriate statistical analysis used?	☒	☐	☐	☐

Overall appraisal: Include ☒ Exclude ☐ Seek further info ☐

Comments (Including reason for exclusion)

- n/a

Critical Appraisal Checklist for Cohort Studies - © JBI, 2020. All rights reserved. JBI grants use of these tools for research purposes only. All other enquiries should be sent to jbisynthesis@adelaide.edu.au.

JBI CRITICAL APPRAISAL CHECKLIST FOR COHORT STUDIES

Reviewer: Froso Sophocleous Date: 2023-11-30

Author: Rampersaud et al. (doi: 10.1182/bloodadvances.2021004634.) Year: 2021 Record Number: AD

	Yes	No	Unclear	Not applicable
1. Were the groups similar and recruited from the same population?	☒	☐	☐	☐
2. Were the exposures measured similarly to assign people to both exposed and unexposed groups?	☒	☐	☐	☐
3. Was the exposure measured in a valid and reliable way?	☒	☐	☐	☐
4. Were confounding factors identified?	☒	☐	☐	☐
5. Were strategies to deal with confounding factors stated?	☒	☐	☐	☐
6. Were the groups/participants free of the outcome at the start of the study (or at the moment of exposure)?	☐	☒	☐	☐
7. Were the outcomes measured in a valid and reliable way?	☒	☐	☐	☐
8. Was the follow up time reported and sufficient to be long enough for outcomes to occur?	☐	☐	☐	☒
9. Was follow up complete, and if not, were the reasons to loss to follow up described and explored?	☐	☐	☐	☒
10. Were strategies to address incomplete follow up utilized?	☐	☐	☐	☒
11. Was appropriate statistical analysis used?	☒	☐	☐	☐

Overall appraisal: Include ☒ Exclude ☐ Seek further info ☐

Comments (Including reason for exclusion)

- n/a

Critical Appraisal Checklist for Cohort Studies - © JBI, 2020. All rights reserved. JBI grants use of these tools for research purposes only. All other enquiries should be sent to jbisynthesis@adelaide.edu.au.

JBI CRITICAL APPRAISAL CHECKLIST FOR COHORT STUDIES

Reviewer: Froso Sophocleous Date: 2023-11-30

Author: Demers et al. (doi: 10.1182/bloodadvances.2020003670) Year: 2021 Record Number: AE

	Yes	No	Unclear	Not applicable
1. Were the groups similar and recruited from the same population?	☐	☐	☐	☒
2. Were the exposures measured similarly to assign people to both exposed and unexposed groups?	☐	☐	☐	☒
3. Was the exposure measured in a valid and reliable way?	☒	☐	☐	☐
4. Were confounding factors identified?	☐	☒	☐	☐
5. Were strategies to deal with confounding factors stated?	☐	☒	☐	☐
6. Were the groups/participants free of the outcome at the start of the study (or at the moment of exposure)?	☐	☐	☐	☒
7. Were the outcomes measured in a valid and reliable way?	☐	☐	☐	☒
8. Was the follow up time reported and sufficient to be long enough for outcomes to occur?	☐	☐	☐	☒
9. Was follow up complete, and if not, were the reasons to loss to follow up described and explored?	☐	☐	☐	☒
10. Were strategies to address incomplete follow up utilized?	☐	☐	☐	☒
11. Was appropriate statistical analysis used?	☒	☐	☐	☐

Overall appraisal: Include ☐ Exclude ☒ Seek further info ☐

Comments (Including reason for exclusion)

- No phenotypic outcome

Critical Appraisal Checklist for Cohort Studies - © JBI, 2020. All rights reserved. JBI grants use of these tools for research purposes only. All other enquiries should be sent to jbisynthesis@adelaide.edu.au.

JBI CRITICAL APPRAISAL CHECKLIST FOR COHORT STUDIES

Reviewer: Froso Sophocleous Date: 2023-11-30

Author: Allard et al. (doi: 10.3324/haematol.2021.278952) Year: 2022 Record Number: AF

	Yes	No	Unclear	Not applicable
1. Were the groups similar and recruited from the same population?	☐	☐	☐	☒
2. Were the exposures measured similarly to assign people to both exposed and unexposed groups?	☐	☐	☐	☒
3. Was the exposure measured in a valid and reliable way?	☒	☐	☐	☐
4. Were confounding factors identified?	☐	☒	☐	☐
5. Were strategies to deal with confounding factors stated?	☐	☒	☐	☐
6. Were the groups/participants free of the outcome at the start of the study (or at the moment of exposure)?	☐	☒	☐	☐
7. Were the outcomes measured in a valid and reliable way?	☒	☐	☐	☐
8. Was the follow up time reported and sufficient to be long enough for outcomes to occur?	☒	☐	☐	☐
9. Was follow up complete, and if not, were the reasons to loss to follow up described and explored?	☐	☐	☒	☐
10. Were strategies to address incomplete follow up utilized?	☐	☐	☒	☐
11. Was appropriate statistical analysis used?	☒	☐	☐	☐

Overall appraisal: Include ☒ Exclude ☐ Seek further info ☐

Comments (Including reason for exclusion)

- n/a

Critical Appraisal Checklist for Cohort Studies - © JBI, 2020. All rights reserved. JBI grants use of these tools for research purposes only. All other enquiries should be sent to jbisynthesis@adelaide.edu.au.

JBI CRITICAL APPRAISAL CHECKLIST FOR COHORT STUDIES

Reviewer: Carsten W Lederer Date: 2023-11-30

Author: Joly et al. (doi: 10.3233/CH-200951) Year: 2021 Record Number: AG

	Yes	No	Unclear	Not applicable
1. Were the groups similar and recruited from the same population?	☒	☐	☐	☐
2. Were the exposures measured similarly to assign people to both exposed and unexposed groups?	☒	☐	☐	☐
3. Was the exposure measured in a valid and reliable way?	☒	☐	☐	☐
4. Were confounding factors identified?	☒	☐	☐	☐
5. Were strategies to deal with confounding factors stated?	☒	☐	☐	☐
6. Were the groups/participants free of the outcome at the start of the study (or at the moment of exposure)?	☐	☒	☐	☐
7. Were the outcomes measured in a valid and reliable way?	☒	☐	☐	☐
8. Was the follow up time reported and sufficient to be long enough for outcomes to occur?	☒	☐	☐	☐
9. Was follow up complete, and if not, were the reasons to loss to follow up described and explored?	☒	☐	☐	☐
10. Were strategies to address incomplete follow up utilized?	☐	☐	☐	☒
11. Was appropriate statistical analysis used?	☒	☐	☐	☐

Overall appraisal: Include ☒ Exclude ☐ Seek further info ☐

Comments (Including reason for exclusion)

- A cohort of 314 unrelated SCA children in four centres in France, were assessed for their α-globin genotype and separated into four non-overlapping groups of similar age and gender distribution, for (1) VOC occurrence, (2) vasculopathy, (3) anaemia and (4) absence of complications. The VOC group was significantly enriched for the -α3.7 alleles ($p<0.001$), the vasculopathy group was significantly depleted of -α3.7 alleles ($p<0.001$).

Critical Appraisal Checklist for Cohort Studies - © JBI, 2020. All rights reserved. JBI grants use of these tools for research purposes only. All other enquiries should be sent to jbisynthesis@adelaide.edu.au.

JBI CRITICAL APPRAISAL CHECKLIST FOR COHORT STUDIES

Reviewer: Froso Sophocleous Date: 2023-11-30

Author: Rumaney et al. (doi: 10.1371/journal.pone.0100516) Year: 2014 Record Number: AH

	Yes	No	Unclear	Not applicable
1. Were the groups similar and recruited from the same population?	☒	☐	☐	☐
2. Were the exposures measured similarly to assign people to both exposed and unexposed groups?	☒	☐	☐	☐
3. Was the exposure measured in a valid and reliable way?	☒	☐	☐	☐
4. Were confounding factors identified?	☒	☐	☐	☐
5. Were strategies to deal with confounding factors stated?	☒	☐	☐	☐
6. Were the groups/participants free of the outcome at the start of the study (or at the moment of exposure)?	☐	☒	☐	☐
7. Were the outcomes measured in a valid and reliable way?	☒	☐	☐	☐
8. Was the follow up time reported and sufficient to be long enough for outcomes to occur?	☐	☐	☐	☒
9. Was follow up complete, and if not, were the reasons to loss to follow up described and explored?	☐	☐	☐	☒
10. Were strategies to address incomplete follow up utilized?	☐	☐	☐	☒
11. Was appropriate statistical analysis used?	☒	☐	☐	☐

Overall appraisal: Include ☒ Exclude ☐ Seek further info ☐

Comments (Including reason for exclusion)

- n/a

Critical Appraisal Checklist for Cohort Studies - © JBI, 2020. All rights reserved. JBI grants use of these tools for research purposes only. All other enquiries should be sent to jbisynthesis@adelaide.edu.au.

JBI CRITICAL APPRAISAL CHECKLIST FOR COHORT STUDIES

Reviewer: __Froso Sophocleous___________________ Date: _2023-12-01 ___________________________

Author: Zhang et al. (doi: 10.2147/JPR.S149958)__ Year: __2018 ______________ Record Number: AI

	Yes	No	Unclear	Not applicable
1. Were the groups similar and recruited from the same population?	☐	☐	☐	☒
2. Were the exposures measured similarly to assign people to both exposed and unexposed groups?	☐	☐	☐	☒
3. Was the exposure measured in a valid and reliable way?	☒	☐	☐	☐
4. Were confounding factors identified?	☒	☐	☐	☐
5. Were strategies to deal with confounding factors stated?	☒	☐	☐	☐
6. Were all groups/participants free of the outcome at the start of the study (or at the moment of exposure)?	☐	☒	☐	☐
7. Were the outcomes measured in a valid and reliable way?	☒	☐	☐	☐
8. Was the follow up time reported and sufficient to be long enough for outcomes to occur?	☐	☐	☐	☒
9. Was follow up complete, and if not, were the reasons to loss to follow up described and explored?	☐	☐	☐	☒
10. Were strategies to address incomplete follow up utilized?	☐	☐	☐	☒
11. Was appropriate statistical analysis used?	☒	☐	☐	☐

Overall appraisal: Include ☒ Exclude ☐ Seek further info ☐

Comments (Including reason for exclusion)

- n/a

Critical Appraisal Checklist for Cohort Studies - © JBI, 2020. All rights reserved. JBI grants use of these tools for research purposes only. All other enquiries should be sent to jbisynthesis@adelaide.edu.au.

JBI CRITICAL APPRAISAL CHECKLIST FOR COHORT STUDIES

Reviewer: Froso Sophocleous Date: 2023-12-04

Author: Armenis et al. (doi: 10.1016/j.dib.2016.11.082) Year: 2016 Record Number: AJ

	Yes	No	Unclear	Not applicable
1. Were the groups similar and recruited from the same population?	X			
2. Were the exposures measured similarly to assign people to both exposed and unexposed groups?	X			
3. Was the exposure measured in a valid and reliable way?	X			
4. Were confounding factors identified?	X			
5. Were strategies to deal with confounding factors stated?		X		
6. Were all groups/participants free of the outcome at the start of the study (or at the moment of exposure)?		X		
7. Were the outcomes measured in a valid and reliable way?	X			
8. Was the follow up time reported and sufficient to be long enough for outcomes to occur?				X
9. Was follow up complete, and if not, were the reasons to loss to follow up described and explored?				X
10. Were strategies to address incomplete follow up utilized?				X
11. Was appropriate statistical analysis used?	X			

Overall appraisal: Include ☒ Exclude ☐ Seek further info ☐

Comments (Including reason for exclusion)

- There are no significant results for relevant phenotypes and the eNOS polymorphisms.
- Significant VOC difference between severe (HbSS, HbS/β^0; n=34) and mild (HbS/β^+, HbS/Lepore; n=45) HbS genotypes, with different gender ratios but similar age ranges across groups. **Inclusion for the effect of β genotypes.**

Critical Appraisal Checklist for Cohort Studies - © JBI, 2020. All rights reserved. JBI grants use of these tools for research purposes only. All other enquiries should be sent to jbisynthesis@adelaide.edu.au.

JBI CRITICAL APPRAISAL CHECKLIST FOR COHORT STUDIES

Reviewer: Froso Sophocleous Date: 2023-12-04

Author: Ozahata et al. (doi: 10.1016/j.jsxm.2019.09.012) Year: 2019 Record Number: AK

	Yes	No	Unclear	Not applicable
1. Were the groups similar and recruited from the same population?	☒	☐	☐	☐
2. Were the exposures measured similarly to assign people to both exposed and unexposed groups?	☒	☐	☐	☐
3. Was the exposure measured in a valid and reliable way?	☒	☐	☐	☐
4. Were confounding factors identified?	☒	☐	☐	☐
5. Were strategies to deal with confounding factors stated?	☒	☐	☐	☐
6. Were all groups/participants free of the outcome at the start of the study (or at the moment of exposure)?	☐	☒	☐	☐
7. Were the outcomes measured in a valid and reliable way?	☒	☐	☐	☐
8. Was the follow up time reported and sufficient to be long enough for outcomes to occur?	☒	☐	☐	☐
9. Was follow up complete, and if not, were the reasons to loss to follow up described and explored?	☒	☐	☐	☐
10. Were strategies to address incomplete follow up utilized?	☐	☐	☐	☒
11. Was appropriate statistical analysis used?	☒	☐	☐	☐

Overall appraisal: Include ☒ Exclude ☐ Seek further info ☐

Comments (Including reason for exclusion)

- n/a

Critical Appraisal Checklist for Cohort Studies - © JBI, 2020. All rights reserved. JBI grants use of these tools for research purposes only. All other enquiries should be sent to jbisynthesis@adelaide.edu.au.

JBI CRITICAL APPRAISAL CHECKLIST FOR COHORT STUDIES

Reviewer: Froso Sophocleous Date: 2023-12-04

Author: Chaturvedi et al. (doi: 10.1182/blood-2017-02-769661) Year: 2017 Record Number: AL

	Yes	No	Unclear	Not applicable
1. Were the groups similar and recruited from the same population?	☒	☐	☐	☐
2. Were the exposures measured similarly to assign people to both exposed and unexposed groups?	☒	☐	☐	☐
3. Was the exposure measured in a valid and reliable way?	☒	☐	☐	☐
4. Were confounding factors identified?	☒	☐	☐	☐
5. Were strategies to deal with confounding factors stated?	☒	☐	☐	☐
6. Were all groups/participants free of the outcome at the start of the study (or at the moment of exposure)?	☐	☒	☐	☐
7. Were the outcomes measured in a valid and reliable way?	☒	☐	☐	☐
8. Was the follow up time reported and sufficient to be long enough for outcomes to occur?	☒	☐	☐	☐
9. Was follow up complete, and if not, were the reasons to loss to follow up described and explored?	☒	☐	☐	☐
10. Were strategies to address incomplete follow up utilized?	☐	☐	☐	☐
11. Was appropriate statistical analysis used?	☒	☐	☐	☐

Overall appraisal: Include ☒ Exclude ☐ Seek further info ☐

Comments (Including reason for exclusion)

- n/a

Critical Appraisal Checklist for Cohort Studies - © JBI, 2020. All rights reserved. JBI grants use of these tools for research purposes only. All other enquiries should be sent to jbisynthesis@adelaide.edu.au.

JBI CRITICAL APPRAISAL CHECKLIST FOR COHORT STUDIES

Reviewer: Carsten W Lederer Date: 2023-12-04

Author: Kucukal et al. (doi: 10.1182/bloodadvances.2020001656) Year: 2020 Record Number: AM

	Yes	No	Unclear	Not applicable
1. Were the groups similar and recruited from the same population?	☐	☐	☐	☒
2. Were the exposures measured similarly to assign people to both exposed and unexposed groups?	☐	☐	☐	☒
3. Was the exposure measured in a valid and reliable way?	☐	☐	☐	☒
4. Were confounding factors identified?	☐	☒	☐	☐
5. Were strategies to deal with confounding factors stated?	☐	☐	☐	☒
6. Were all groups/participants free of the outcome at the start of the study (or at the moment of exposure)?	☐	☐	☐	☒
7. Were the outcomes measured in a valid and reliable way?	☒	☐	☐	☐
8. Was the follow up time reported and sufficient to be long enough for outcomes to occur?	☐	☐	☐	☒
9. Was follow up complete, and if not, were the reasons to loss to follow up described and explored?	☐	☐	☐	☒
10. Were strategies to address incomplete follow up utilized?	☐	☐	☐	☒
11. Was appropriate statistical analysis used?	☒	☐	☐	☐

Overall appraisal: Include ☐ Exclude ☒ Seek further info ☐

Comments (Including reason for exclusion)

- *In vitro* study correlating adhesion behaviour with *in vivo* phenotypes, but without reference to specific variants and not investigating the specific phenotypes of interest for this study. Included only as supportive evidence for other ICAM1-related data in this review.

Critical Appraisal Checklist for Cohort Studies - © JBI, 2020. All rights reserved. JBI grants use of these tools for research purposes only. All other enquiries should be sent to jbisynthesis@adelaide.edu.au.

JBI CRITICAL APPRAISAL CHECKLIST FOR COHORT STUDIES

Reviewer: Froso Sophocleous Date: 2023-12-06

Author: Wonkam et al. (doi: 10.1111/bjh.15011) Year: 2018 Record Number: AN

	Yes	No	Unclear	Not applicable
1. Were the groups similar and recruited from the same population?	☐	☐	☐	☒
2. Were the exposures measured similarly to assign people to both exposed and unexposed groups?	☐	☐	☐	☒
3. Was the exposure measured in a valid and reliable way?	☒	☐	☐	☐
4. Were confounding factors identified?	☒	☐	☒	☐
5. Were strategies to deal with confounding factors stated?	☒	☐	☐	☐
6. Were all groups/participants free of the outcome at the start of the study (or at the moment of exposure)?	☐	☒	☐	☐
7. Were the outcomes measured in a valid and reliable way?	☒	☐	☐	☐
8. Was the follow up time reported and sufficient to be long enough for outcomes to occur?	☐	☐	☐	☒
9. Was follow up complete, and if not, were the reasons to loss to follow up described and explored?	☐	☐	☐	☒
10. Were strategies to address incomplete follow up utilized?	☐	☐	☐	☒
11. Was appropriate statistical analysis used?	☒	☐	☐	☐

Overall appraisal: Include ☒ Exclude ☐ Seek further info ☐

Comments (Including reason for exclusion)

- There is a single group of 436 SCDs which is well described.
- It is mentioned in limitations that there was some loss during follow up or due to death.
- This is a hypothesis-tested study, but there is no regression to identify the size and direction of the effect. Nevertheless, there was no significant association to further test for the effect.

Critical Appraisal Checklist for Cohort Studies - © JBI, 2020. All rights reserved. JBI grants use of these tools for research purposes only. All other enquiries should be sent to jbisynthesis@adelaide.edu.au.

JBI CRITICAL APPRAISAL CHECKLIST FOR COHORT STUDIES

Reviewer: Carsten W Lederer Date: 2023-12-03

Author: Kumar et al. (doi: 10.1007/s00277-020-04381-z) Year: 2021 Record Number: AO

	Yes	No	Unclear	Not applicable
1. Were the groups similar and recruited from the same population?	☐	☐	☐	☒
2. Were the exposures measured similarly to assign people to both exposed and unexposed groups?	☒	☐	☐	☐
3. Was the exposure measured in a valid and reliable way?	☒	☐	☐	☐
4. Were confounding factors identified?	☒	☐	☐	☐
5. Were strategies to deal with confounding factors stated?	☐	☐	☒	☐
6. Were all groups/participants free of the outcome at the start of the study (or at the moment of exposure)?	☐	☒	☐	☐
7. Were the outcomes measured in a valid and reliable way?	☒	☐	☐	☐
8. Was the follow up time reported and sufficient to be long enough for outcomes to occur?	☒	☐	☐	☐
9. Was follow up complete, and if not, were the reasons to loss to follow up described and explored?	☒	☐	☐	☐
10. Were strategies to address incomplete follow up utilized?	☐	☐	☐	☒
11. Was appropriate statistical analysis used?	☒	☐	☐	☐

Overall appraisal: Include ☐ Exclude ☒ Seek further info ☐

Comments (Including reason for exclusion)

- Indian paediatric cohort of 118 with 5-year follow-up (3-10 yrs), retrospective medical records, PCR-RFLP for the XmnI polymorphism and Sanger-based sequencing of KLF1. No significant differences for XmnI and KLF1 variants were detected for any of the parameters tested. Inclusion of the negative finding in the text only.

Critical Appraisal Checklist for Cohort Studies - © JBI, 2020. All rights reserved. JBI grants use of these tools for research purposes only. All other enquiries should be sent to jbisynthesis@adelaide.edu.au.

JBI CRITICAL APPRAISAL CHECKLIST FOR COHORT STUDIES

Reviewer: Froso Sophocleous Date: 2023-12-07

Author: Wang et al. (doi: 10.1182/bloodadvances.2021006668) Year: 2022 Record Number: AP

	Yes	No	Unclear	Not applicable
1. Were the groups similar and recruited from the same population?	☒	☐	☐	☐
2. Were the exposures measured similarly to assign people to both exposed and unexposed groups?	☐	☐	☐	☒
3. Was the exposure measured in a valid and reliable way?	☒	☐	☐	☐
4. Were confounding factors identified?	☐	☒	☐	☐
5. Were strategies to deal with confounding factors stated?	☐	☒	☐	☐
6. Were all groups/participants free of the outcome at the start of the study (or at the moment of exposure)?	☐	☒	☐	☐
7. Were the outcomes measured in a valid and reliable way?	☒	☐	☐	☐
8. Was the follow up time reported and sufficient to be long enough for outcomes to occur?	☐	☐	☐	☒
9. Was follow up complete, and if not, were the reasons to loss to follow up described and explored?	☐	☐	☐	☒
10. Were strategies to address incomplete follow up utilized?	☐	☐	☐	☒
11. Was appropriate statistical analysis used?	☒	☐	☐	☐

Overall appraisal: Include ☒ Exclude ☐ Seek further info ☐

Comments (Including reason for exclusion)

- There are 3 different cohorts of patients from different sources (no controls). One cohort used to measure allelic imbalance (or none) in a control population
- Groupwise comparisons performed within cohorts (paediatric, US; adult, UK; control from three different trials)

Critical Appraisal Checklist for Cohort Studies - © JBI, 2020. All rights reserved. JBI grants use of these tools for research purposes only. All other enquiries should be sent to jbisynthesis@adelaide.edu.au.

JBI CRITICAL APPRAISAL CHECKLIST FOR COHORT STUDIES

Reviewer: Carsten W Lederer Date: 2023-12-07

Author: Sabrie et al. (doi: 10.1080/03630269.2018.1529602) Year: 2018 Record Number: AQ

	Yes	No	Unclear	Not applicable
1. Were the groups similar and recruited from the same population?	☒	☐	☐	☐
2. Were the exposures measured similarly to assign people to both exposed and unexposed groups?	☒	☐	☐	☐
3. Was the exposure measured in a valid and reliable way?	☒	☐	☐	☐
4. Were confounding factors identified?	☒	☐	☐	☐
5. Were strategies to deal with confounding factors stated?	☒	☐	☐	☐
6. Were all groups/participants free of the outcome at the start of the study (or at the moment of exposure)?	☐	☒	☐	☐
7. Were the outcomes measured in a valid and reliable way?	☒	☐	☐	☐
8. Was the follow up time reported and sufficient to be long enough for outcomes to occur?	☒	☐	☐	☐
9. Was follow up complete, and if not, were the reasons to loss to follow up described and explored?	☒	☐	☐	☐
10. Were strategies to address incomplete follow up utilized?	☐	☐	☐	☒
11. Was appropriate statistical analysis used?	☒	☐	☐	☐

Overall appraisal: Include ☐ Exclude ☒ Seek further info ☐

Comments (Including reason for exclusion)

- A cohort of 58 SCD patients (18-60 yrs) was quantified for the number of hospitalizations and frequency and quantity of morphine administration, in relation to α-thalassemia, UGT2B7 and ABCB1 genotypes. No significant associations were found for parameters relevant for this study or morphine use. Inclusion in the text only, for the relevance of environmental factors on the assessment of pain phenomena in SCD.

Critical Appraisal Checklist for Cohort Studies - © JBI, 2020. All rights reserved. JBI grants use of these tools for research purposes only. All other enquiries should be sent to jbisynthesis@adelaide.edu.au.

JBI CRITICAL APPRAISAL CHECKLIST FOR COHORT STUDIES

Reviewer: Froso Sophocleous Date: 2023-12-08

Author: Sokkar et al. (https://doi.org/10.1016/j.genrep.2022.101595) Year: 2022 Record Number: AR

	Yes	No	Unclear	Not applicable
1. Were the groups similar and recruited from the same population?	☒	☐	☐	☐
2. Were the exposures measured similarly to assign people to both exposed and unexposed groups?	☒	☐	☐	☐
3. Was the exposure measured in a valid and reliable way?	☒	☐	☐	☐
4. Were confounding factors identified?	☐	☒	☐	☐
5. Were strategies to deal with confounding factors stated?	☐	☒	☐	☐
6. Were all groups/participants free of the outcome at the start of the study (or at the moment of exposure)?	☐	☒	☐	☐
7. Were the outcomes measured in a valid and reliable way?	☒	☐	☐	☐
8. Was the follow up time reported and sufficient to be long enough for outcomes to occur?	☐	☐	☐	☒
9. Was follow up complete, and if not, were the reasons to loss to follow up described and explored?	☐	☐	☐	☒
10. Were strategies to address incomplete follow up utilized?	☐	☐	☐	☒
11. Was appropriate statistical analysis used?	☒	☐	☐	☐

Overall appraisal: Include ☒ Exclude ☐ Seek further info ☐

Comments (Including reason for exclusion)

- n/a

Critical Appraisal Checklist for Cohort Studies - © JBI, 2020. All rights reserved. JBI grants use of these tools for research purposes only. All other enquiries should be sent to jbisynthesis@adelaide.edu.au.

JBI CRITICAL APPRAISAL CHECKLIST FOR COHORT STUDIES

Reviewer: Froso Sophocleous Date: 2023-12-08

Author: Abdulwahab et al. (DOI: 10.1038/s41598-021-01702-8) Year: 2021 Record Number: AS

	Yes	No	Unclear	Not applicable
1. Were the groups similar and recruited from the same population?	☒	☐	☐	☐
2. Were the exposures measured similarly to assign people to both exposed and unexposed groups?	☒	☐	☐	☐
3. Was the exposure measured in a valid and reliable way?	☒	☐	☐	☐
4. Were confounding factors identified?	☐	☒	☐	☐
5. Were strategies to deal with confounding factors stated?	☐	☒	☐	☐
6. Were all groups/participants free of the outcome at the start of the study (or at the moment of exposure)?	☐	☒	☐	☐
7. Were the outcomes measured in a valid and reliable way?	☒	☐	☐	☐
8. Was the follow up time reported and sufficient to be long enough for outcomes to occur?	☐	☐	☐	☒
9. Was follow up complete, and if not, were the reasons to loss to follow up described and explored?	☐	☐	☐	☒
10. Were strategies to address incomplete follow up utilized?	☐	☐	☐	☒
11. Was appropriate statistical analysis used?	☒	☐	☐	☐

Overall appraisal: Include ☒ Exclude ☐ Seek further info ☐

Comments (Including reason for exclusion)

- n/a

Critical Appraisal Checklist for Cohort Studies - © JBI, 2020. All rights reserved. JBI grants use of these tools for research purposes only. All other enquiries should be sent to jbisynthesis@adelaide.edu.au.

JBI CRITICAL APPRAISAL CHECKLIST FOR COHORT STUDIES

Reviewer: Froso Sophocleous Date: 2023-12-06

Author: Shiba et al. (doi: 10.2350/14-03-1452-OA.1) Year: 2014 Record Number: AT

	Yes	No	Unclear	Not applicable
1. Were the groups similar and recruited from the same population?	☒	☐	☐	☐
2. Were the exposures measured similarly to assign people to both exposed and unexposed groups?	☒	☐	☐	☐
3. Was the exposure measured in a valid and reliable way?	☒	☐	☐	☐
4. Were confounding factors identified?	☒	☐	☐	☐
5. Were strategies to deal with confounding factors stated?	☐	☒	☐	☐
6. Were all groups/participants free of the outcome at the start of the study (or at the moment of exposure)?	☐	☒	☐	☐
7. Were the outcomes measured in a valid and reliable way?	☒	☐	☐	☐
8. Was the follow up time reported and sufficient to be long enough for outcomes to occur?	☐	☐	☐	☒
9. Was follow up complete, and if not, were the reasons to loss to follow up described and explored?	☐	☐	☐	☒
10. Were strategies to address incomplete follow up utilized?	☐	☐	☐	☒
11. Was appropriate statistical analysis used?	☒	☐	☐	☐

Overall appraisal: Include ☒ Exclude ☐ Seek further info ☐

Comments (Including reason for exclusion)

- n/a

Critical Appraisal Checklist for Cohort Studies - © JBI, 2020. All rights reserved. JBI grants use of these tools for research purposes only. All other enquiries should be sent to jbisynthesis@adelaide.edu.au.

JBI CRITICAL APPRAISAL CHECKLIST FOR COHORT STUDIES

Reviewer: Froso Sophocleous Date: 2023-12-08

Author: Zhang et al. (doi: 10.1002/ajh.25691) Year: 2020 Record Number: AU

	Yes	No	Unclear	Not applicable
1. Were the groups similar and recruited from the same population?	☐	☒	☐	☐
2. Were the exposures measured similarly to assign people to both exposed and unexposed groups?	☐	☐	☐	☒
3. Was the exposure measured in a valid and reliable way?	☒	☐	☐	☐
4. Were confounding factors identified?	☐	☐	☒	☐
5. Were strategies to deal with confounding factors stated?	☐	☒	☐	☐
6. Were all groups/participants free of the outcome at the start of the study (or at the moment of exposure)?	☐	☒	☐	☐
7. Were the outcomes measured in a valid and reliable way?	☒	☐	☐	☐
8. Was the follow up time reported and sufficient to be long enough for outcomes to occur?	☐	☐	☐	☒
9. Was follow up complete, and if not, were the reasons to loss to follow up described and explored?	☐	☐	☐	☒
10. Were strategies to address incomplete follow up utilized?	☐	☐	☐	☒
11. Was appropriate statistical analysis used?	☐	☐	☒	☐

Overall appraisal: Include ☒ Exclude ☐ Seek further info ☐

Comments (Including reason for exclusion)

- Multiple cohorts (cross-sectional and profiling cohorts) are not described, neither in the text nor at the supplementary material.
- It is mentioned that the association was analysed using logistic linear regression adjusted for age, gender and clinical sites, but the statistics are not shown.
- There are no statistics as part of the methods.

Critical Appraisal Checklist for Cohort Studies - © JBI, 2020. All rights reserved. JBI grants use of these tools for research purposes only. All other enquiries should be sent to jbisynthesis@adelaide.edu.au.

JBI CRITICAL APPRAISAL CHECKLIST FOR COHORT STUDIES

Reviewer: Froso Sophocleous Date: 2023-12-12

Author: Rosenberg et al. (doi: 10.1016/j.jaapos.2010.11.014) Year: 2011 Record Number: AV

	Yes	No	Unclear	Not applicable
1. Were the groups similar and recruited from the same population?	☒	☐	☐	☐
2. Were the exposures measured similarly to assign people to both exposed and unexposed groups?	☒	☐	☐	☐
3. Was the exposure measured in a valid and reliable way?	☒	☐	☐	☐
4. Were confounding factors identified?	☒	☐	☐	☐
5. Were strategies to deal with confounding factors stated?	☐	☒	☐	☐
6. Were all groups/participants free of the outcome at the start of the study (or at the moment of exposure)?	☐	☒	☐	☐
7. Were the outcomes measured in a valid and reliable way?	☒	☐	☐	☐
8. Was the follow up time reported and sufficient to be long enough for outcomes to occur?	☐	☐	☐	☒
9. Was follow up complete, and if not, were the reasons to loss to follow up described and explored?	☐	☐	☐	☒
10. Were strategies to address incomplete follow up utilized?	☐	☐	☐	☒
11. Was appropriate statistical analysis used?	☐	☒	☐	☐

Overall appraisal: Include ☒ Exclude ☐ Seek further info ☐

Comments (Including reason for exclusion)

- Subgroups poorly explained in the text. Groups undergoing groupwise statistical analyses need to be inferred from the text. By dividing SR vs non-SR, the groups that are compared are approximately similar and from the same population, but are highly heterogeneous by each including HbSS, HbSβ$^{+/0}$ and HbSC
- Not significant: G6PD deficiency was more common in patients with retinopathy, although this was not statistically significant (OR, 4.20; p 5 0.054).
- Statistical analyses were not explained at all and statistical help merely acknowledged. Absence of multiple-testing correction and potential biases are acknowledged.
- Confounding factors were stated (beta-locus genotypes) but not addressed for statistical analyses. Any analysis results are merely indicative, because no error correction was applied and p values are already quite high (lowest p: SR vs non-SR: Male, 0.004)

Critical Appraisal Checklist for Cohort Studies - © JBI, 2020. All rights reserved. JBI grants use of these tools for research purposes only. All other enquiries should be sent to jbisynthesis@adelaide.edu.au.

JBI CRITICAL APPRAISAL CHECKLIST FOR COHORT STUDIES

Reviewer: Carsten W Lederer Date: 2023-12-12

Author: Filho et al. (DOI: 10.5581/1516-8484.20120049) Year: 2012 Record Number: AW

	Yes	No	Unclear	Not applicable
1. Were the groups similar and recruited from the same population?	☒	☐	☐	☐
2. Were the exposures measured similarly to assign people to both exposed and unexposed groups?	☒	☐	☐	☐
3. Was the exposure measured in a valid and reliable way?	☒	☐	☐	☐
4. Were confounding factors identified?	☒	☐	☐	☐
5. Were strategies to deal with confounding factors stated?	☒	☐	☐	☐
6. Were all groups/participants free of the outcome at the start of the study (or at the moment of exposure)?	☐	☒	☐	☐
7. Were the outcomes measured in a valid and reliable way?	☒	☐	☐	☐
8. Was the follow up time reported and sufficient to be long enough for outcomes to occur?	☒	☐	☐	☐
9. Was follow up complete, and if not, were the reasons to loss to follow up described and explored?	☒	☐	☐	☐
10. Were strategies to address incomplete follow up utilized?	☐	☐	☐	☒
11. Was appropriate statistical analysis used?	☒	☐	☐	☐

Overall appraisal: Include ☐ Exclude ☒ Seek further info ☐

Comments (Including reason for exclusion)

- Brazilian paediatric cohort study on 79 HbSS and 17 HbSC patients, with groupwise comparisons for age groups, gender, β- and α-globin genotype, β haplotype. No significant results were obtained for specific variants for the conditions of interest. Inclusion in the text for information about β-globin genotypes and haplotypes.

Critical Appraisal Checklist for Cohort Studies - © JBI, 2020. All rights reserved. JBI grants use of these tools for research purposes only. All other enquiries should be sent to jbisynthesis@adelaide.edu.au.

JBI CRITICAL APPRAISAL CHECKLIST FOR COHORT STUDIES

Reviewer: Froso Sophocleous Date: 2023-12-12

Author: Duckworth et al. (DOI: 10.1002/ppul.20582) Year: 2007 Record Number: AX

	Yes	No	Unclear	Not applicable
1. Were the groups similar and recruited from the same population?	X			
2. Were the exposures measured similarly to assign people to both exposed and unexposed groups?	X			
3. Was the exposure measured in a valid and reliable way?	X			
4. Were confounding factors identified?	X			
5. Were strategies to deal with confounding factors stated?	X			
6. Were all groups/participants free of the outcome at the start of the study (or at the moment of exposure)?		X		
7. Were the outcomes measured in a valid and reliable way?			X	
8. Was the follow up time reported and sufficient to be long enough for outcomes to occur?				X
9. Was follow up complete, and if not, were the reasons to loss to follow up described and explored?				X
10. Were strategies to address incomplete follow up utilized?				X
11. Was appropriate statistical analysis used?	X			

Overall appraisal: Include [X] Exclude [ ] Seek further info [ ]

Comments (Including reason for exclusion)

- The effects of the covariates in the associations were explored by logistic regression analysis including age and sex parameters. Data are not shown for non-association of age with ACS episodes, and of age or gender contribution to high (7/8)-frequency (above 7 times) ACS with asthma. Groupwise comparisons for ACS vs non-ACS with the NOS3 variant are undertaken also in sub-populations to address potentially confounding factors (asthma, non-asthma, male, female), but the data are not shown.
- Confounding factors were not explored to exclude false-positive detection of statistical relationships, but to reduce intra-group variation in groupwise comparisons.
- Diagnosis of ACS and asthma use what are in part overlapping parameters (cough, hypoxia, dyspnoea), so that their distinction might not be clear-cut. Question 6 is answered in relation to ACS, while for question 7, the overlap between asthma and ACS diagnosis but also the (apparent) exclusion of ACS by absence of either cough, hypoxemia or dyspnoea are problematic. The description of one (ACS) being a potential consequence of the other (asthma) seems doubtful, but that is not a core aspect of the paper from the genetic-modifier perspective.
- For questions 8 to 10, follow-up is not the appropriate term; observation of ACS was recorded for first life-time occurrence for each patient.

Critical Appraisal Checklist for Cohort Studies - © JBI, 2020. All rights reserved. JBI grants use of these tools for research purposes only. All other enquiries should be sent to jbisynthesis@adelaide.edu.au.

JBI CRITICAL APPRAISAL CHECKLIST FOR COHORT STUDIES

Reviewer: __Froso Sophocleous__________________ Date: _2023-12-12 ___________________________

Author: Al-Subaie et al. (doi: 10.1111/j.1600-0609.2009.01339.x) ___Year: _2009 Record Number: AY

	Yes	No	Unclear	Not applicable
1. Were the groups similar and recruited from the same population?	☒	☐	☐	☐
2. Were the exposures measured similarly to assign people to both exposed and unexposed groups?	☒	☐	☐	☐
3. Was the exposure measured in a valid and reliable way?	☒	☐	☐	☐
4. Were confounding factors identified?	☒	☐	☐	☐
5. Were strategies to deal with confounding factors stated?	☒	☐	☐	☐
6. Were all groups/participants free of the outcome at the start of the study (or at the moment of exposure)?	☐	☒	☐	☐
7. Were the outcomes measured in a valid and reliable way?	☒	☐	☐	☐
8. Was the follow up time reported and sufficient to be long enough for outcomes to occur?	☐	☐	☐	☒
9. Was follow up complete, and if not, were the reasons to loss to follow up described and explored?	☐	☐	☐	☒
10. Were strategies to address incomplete follow up utilized?	☐	☐	☐	☒
11. Was appropriate statistical analysis used?	☒	☐	☐	☐

Overall appraisal: Include ☒ Exclude ☐ Seek further info ☐

Comments (Including reason for exclusion)

- n/a

Critical Appraisal Checklist for Cohort Studies - © JBI, 2020. All rights reserved. JBI grants use of these tools for research purposes only. All other enquiries should be sent to jbisynthesis@adelaide.edu.au.

JBI CRITICAL APPRAISAL CHECKLIST FOR COHORT STUDIES

Reviewer: Carsten W Lederer Date: 2023-12-14

Author: Alsultan et al. (doi: 10.1097/MPH.0b013e3182422844.) Year: 2012 Record Number: AZ

	Yes	No	Unclear	Not applicable
1. Were the groups similar and recruited from the same population?	☒	☐	☐	☐
2. Were the exposures measured similarly to assign people to both exposed and unexposed groups?	☒	☐	☐	☐
3. Was the exposure measured in a valid and reliable way?	☒	☐	☐	☐
4. Were confounding factors identified?	☒	☐	☐	☐
5. Were strategies to deal with confounding factors stated?	☒	☐	☐	☐
6. Were all groups/participants free of the outcome at the start of the study (or at the moment of exposure)?	☐	☒	☐	☐
7. Were the outcomes measured in a valid and reliable way?	☒	☐	☐	☐
8. Was the follow up time reported and sufficient to be long enough for outcomes to occur?	☒	☐	☐	☐
9. Was follow up complete, and if not, were the reasons to loss to follow up described and explored?	☒	☐	☐	☐
10. Were strategies to address incomplete follow up utilized?	☒	☐	☐	☐
11. Was appropriate statistical analysis used?	☒	☐	☐	☐

Overall appraisal: Include ☐ Exclude ☒ Seek further info ☐

Comments (Including reason for exclusion)

- Saudi Arabian cohort of 179 HbSS, HbSβ0, and HbSβ+ patients, analyzed by multivariate analysis for odds ratios for the occurrence of key patient parameters with different disease manifestations. Significant findings did not relate to specific variants but were included here in the text only, for the role of the α-globin genotype.

Critical Appraisal Checklist for Cohort Studies - © JBI, 2020. All rights reserved. JBI grants use of these tools for research purposes only. All other enquiries should be sent to jbisynthesis@adelaide.edu.au.

JBI CRITICAL APPRAISAL CHECKLIST FOR CASE REPORTS

Reviewer: __Carsten W Lederer___________________ Date: _2024-12-03 __________________________

Author: Patel et al. (doi: 10.1007/s00277-010-1014-1.)__________Year: 2011_ Record Number: BA

	Yes	No	Unclear	Not applicable
1. Were patient's demographic characteristics clearly described?	X			
2. Was the patient's history clearly described and presented as a timeline?		X		
3. Was the current clinical condition of the patient on presentation clearly described?	X			
4. Were diagnostic tests or assessment methods and the results clearly described?	X			
5. Was the intervention(s) or treatment procedure(s) clearly described?	X			
6. Was the post-intervention clinical condition clearly described?				X
7. Were adverse events (harms) or unanticipated events identified and described?				X
8. Does the case report provide takeaway lessons?			X	

Overall appraisal: Include ☐ Exclude ☒ Seek further info ☐

Comments (Including reason for exclusion)

- Brief case report of an Indian HbSC sibling couple, with the index case a 25-yo female of mild phenotype and her brother a 35-yo with more severe disease course, despite shared αα/αα and XmnI (+/+) genotypes. Included in the text only to illustrate diversity of disease severity for same-β-genotype individuals.

Critical Appraisal Checklist for Cohort Studies - © JBI, 2020. All rights reserved. JBI grants use of these tools for research purposes only. All other enquiries should be sent to jbisynthesis@adelaide.edu.au.

JBI CRITICAL APPRAISAL CHECKLIST FOR CASE REPORTS

Reviewer: Carsten W Lederer Date: 2024-12-03

Author: Ustun et al. (doi: 10.1159/000067273.) Year: 2003 Record Number: BC

	Yes	No	Unclear	Not applicable
1. Were patient's demographic characteristics clearly described?	☒	☐	☐	☐
2. Was the patient's history clearly described and presented as a timeline?	☐	☒	☐	☐
3. Was the current clinical condition of the patient on presentation clearly described?	☒	☐	☐	☐
4. Were diagnostic tests or assessment methods and the results clearly described?	☒	☐	☐	☐
5. Was the intervention(s) or treatment procedure(s) clearly described?	☒	☐	☐	☐
6. Was the post-intervention clinical condition clearly described?	☐	☐	☐	☒
7. Were adverse events (harms) or unanticipated events identified and described?	☐	☐	☐	☒
8. Does the case report provide takeaway lessons?	☐	☒	☐	☐

Overall appraisal: Include ☐ Exclude ☒ Seek further info ☐

Comments (Including reason for exclusion)

- The case report for a 46-yo African-American male confirms the HbS mutation by cDNA sequencing and assumes from the absence of other detectable globin variants by cDNA sequencing and from microscopic observation that this is a case of combined carrier status for SCD and spheric hemocytosis. The latter is not shown molecularly and the former is not proven fully by today's sequencing standards.

Critical Appraisal Checklist for Cohort Studies - © JBI, 2020. All rights reserved. JBI grants use of these tools for research purposes only. All other enquiries should be sent to jbisynthesis@adelaide.edu.au.

JBI CRITICAL APPRAISAL CHECKLIST FOR COHORT STUDIES

Reviewer: Froso Sophocleous Date: 2023-12-19

Author: Bean et al. (doi: 10.1182/blood-2011-06-361642) Year: 2012 Record Number: BD

	Yes	No	Unclear	Not applicable
1. Were the groups similar and recruited from the same population?	☐	☐	☐	☒
2. Were the exposures measured similarly to assign people to both exposed and unexposed groups?	☐	☐	☐	☒
3. Was the exposure measured in a valid and reliable way?	☒	☐	☐	☐
4. Were confounding factors identified?	☒	☐	☐	☐
5. Were strategies to deal with confounding factors stated?	☒	☐	☐	☐
6. Were all groups/participants free of the outcome at the start of the study (or at the moment of exposure)?	☐	☒	☐	☐
7. Were the outcomes measured in a valid and reliable way?	☒	☐	☐	☐
8. Was the follow up time reported and sufficient to be long enough for outcomes to occur?	☐	☐	☐	☒
9. Was follow up complete, and if not, were the reasons to loss to follow up described and explored?	☐	☐	☐	☒
10. Were strategies to address incomplete follow up utilized?	☐	☐	☐	☒
11. Was appropriate statistical analysis used?	☒	☐	☐	☐

Overall appraisal: Include ☒ Exclude ☐ Seek further info ☐

Comments (Including reason for exclusion)

- A single group study with no controls.

Critical Appraisal Checklist for Cohort Studies - © JBI, 2020. All rights reserved. JBI grants use of these tools for research purposes only. All other enquiries should be sent to jbisynthesis@adelaide.edu.au.

JBI CRITICAL APPRAISAL CHECKLIST FOR COHORT STUDIES

Reviewer: Froso Sophocleous Date: 2023-12-20

Author: Mahdi et al. (DOI 10.1007/s00277-012-1474-6) Year: 2012 Record Number: BE

	Yes	No	Unclear	Not applicable
1. Were the groups similar and recruited from the same population?	☒	☐	☐	☐
2. Were the exposures measured similarly to assign people to both exposed and unexposed groups?	☒	☐	☐	☐
3. Was the exposure measured in a valid and reliable way?	☒	☐	☐	☐
4. Were confounding factors identified?	☒	☐	☐	☐
5.				
6. Were strategies to deal with confounding factors stated?	☒	☐	☐	☐
7. Were all groups/participants free of the outcome at the start of the study (or at the moment of exposure)?	☐	☒	☐	☐
8. Were the outcomes measured in a valid and reliable way?	☒	☐	☐	☐
9. Was the follow up time reported and sufficient to be long enough for outcomes to occur?	☐	☐	☐	☒
10. Was follow up complete, and if not, were the reasons to loss to follow up described and explored?	☐	☐	☐	☒
11. Were strategies to address incomplete follow up utilized?	☐	☐	☐	☒
12. Was appropriate statistical analysis used?	☒	☐	☐	☐

Overall appraisal: Include ☒ Exclude ☐ Seek further info ☐

Comments (Including reason for exclusion)

- n/a

Critical Appraisal Checklist for Cohort Studies - © JBI, 2020. All rights reserved. JBI grants use of these tools for research purposes only. All other enquiries should be sent to jbisynthesis@adelaide.edu.au.

JBI CRITICAL APPRAISAL CHECKLIST FOR COHORT STUDIES

Reviewer: Froso Sophocleous Date: 2023-12-20

Author: Chaar et al. (pubmed.ncbi.nlm.nih.gov/16956834/) Year: 2006 Record Number: BF

	Yes	No	Unclear	Not applicable
1. Were the groups similar and recruited from the same population?	☐	☐	☐	☒
2. Were the exposures measured similarly to assign people to both exposed and unexposed groups?	☐	☐	☐	☒
3. Was the exposure measured in a valid and reliable way?	☒	☐	☐	☐
4. Were confounding factors identified?	☒	☐	☐	☐
5. Were strategies to deal with confounding factors stated?	☒	☐	☐	☐
6. Were all groups/participants free of the outcome at the start of the study (or at the moment of exposure)?	☐	☒	☐	☐
7. Were the outcomes measured in a valid and reliable way?	☒	☐	☐	☐
8. Was the follow up time reported and sufficient to be long enough for outcomes to occur?	☒	☐	☐	☐
9. Was follow up complete, and if not, were the reasons to loss to follow up described and explored?	☐	☐	☒	☐
10. Were strategies to address incomplete follow up utilized?	☐	☐	☒	☐
11. Was appropriate statistical analysis used?	☒	☐	☐	☐

Overall appraisal: Include ☒ Exclude ☐ Seek further info ☐

Comments (Including reason for exclusion)

- In this study there is only one group of SS children.

Critical Appraisal Checklist for Cohort Studies - © JBI, 2020. All rights reserved. JBI grants use of these tools for research purposes only. All other enquiries should be sent to jbisynthesis@adelaide.edu.au.

JBI CRITICAL APPRAISAL CHECKLIST FOR COHORT STUDIES

Reviewer: Froso Sophocleous Date: 2023-12-20

Author: Nishank et al. (DOI:10.1111/ejh.12190) Year: 2013 Record Number: BG

	Yes	No	Unclear	Not applicable
1. Were the groups similar and recruited from the same population?	☒	☐	☐	☐
2. Were the exposures measured similarly to assign people to both exposed and unexposed groups?	☒	☐	☐	☐
3. Was the exposure measured in a valid and reliable way?	☒	☐	☐	☐
4. Were confounding factors identified?	☐	☒	☐	☐
5. Were strategies to deal with confounding factors stated?	☐	☒	☐	☐
6. Were all groups/participants free of the outcome at the start of the study (or at the moment of exposure)?	☐	☒	☐	☐
7. Were the outcomes measured in a valid and reliable way?	☒	☐	☐	☐
8. Was the follow up time reported and sufficient to be long enough for outcomes to occur?	☒	☐	☐	☐
9. Was follow up complete, and if not, were the reasons to loss to follow up described and explored?	☐	☐	☒	☐
10. Were strategies to address incomplete follow up utilized?	☐	☐	☒	☐
11. Was appropriate statistical analysis used?	☐	☒	☐	☐

Overall appraisal: Include ☒ Exclude ☐ Seek further info ☐

Comments (Including reason for exclusion)

- It is unclear from where the chest pain, bone joint pain, abdominal pain, fatigue, fever, splenomegaly, history of frequent requirement of blood transfusion per year, as well as early age of onset of manifestation of clinical symptoms were derived.
- It seems follow-up is lifetime follow-up of summary data, measurement a one-off measurement.
- Statistics to do not give criteria/tests for choice of parametric tests. No error correction was applied for multiple p tests for the same population, and tests were performed for (somewhat arbitrary) age groups as sub-populations, which could have been addressed by more appropriate statistical tests. However, for MTHFR the lowest-p results would likely have statistical significance also after application of error correction. This is likely in line with biological significance, such as fever, splenomegaly, transfusion requirements and age of onset.
- The follow up procedure is not explained.

Critical Appraisal Checklist for Cohort Studies - © JBI, 2020. All rights reserved. JBI grants use of these tools for research purposes only. All other enquiries should be sent to jbisynthesis@adelaide.edu.au.

JBI CRITICAL APPRAISAL CHECKLIST FOR COHORT STUDIES

Reviewer: Carsten W Lederer Date: 2024-12-03

Author: Jain et al. (PMID: 24135175) Year: 2013 Record Number: BH

	Yes	No	Unclear	Not applicable
1. Were the groups similar and recruited from the same population?	☐	☐	☐	☒
2. Were the exposures measured similarly to assign people to both exposed and unexposed groups?	☐	☐	☐	☒
3. Was the exposure measured in a valid and reliable way?	☒	☐	☐	☐
4. Were confounding factors identified?	☐	☐	☒	☐
5. Were strategies to deal with confounding factors stated?	☐	☒	☐	☐
6. Were all groups/participants free of the outcome at the start of the study (or at the moment of exposure)?	☐	☐	☐	☒
7. Were the outcomes measured in a valid and reliable way?	☒	☐	☐	☐
8. Was the follow up time reported and sufficient to be long enough for outcomes to occur?	☒	☐	☐	☐
9. Was follow up complete, and if not, were the reasons to loss to follow up described and explored?	☒	☐	☐	☐
10. Were strategies to address incomplete follow up utilized?	☐	☐	☐	☒
11. Was appropriate statistical analysis used?	☒	☐	☐	☐

Overall appraisal: Include ☐ Exclude ☒ Seek further info ☐

Comments (Including reason for exclusion)

- No genotypes or variants were reported in this study.

Critical Appraisal Checklist for Cohort Studies - © JBI, 2020. All rights reserved. JBI grants use of these tools for research purposes only. All other enquiries should be sent to jbisynthesis@adelaide.edu.au.

JBI CRITICAL APPRAISAL CHECKLIST FOR COHORT STUDIES

Reviewer: __Carsten W Lederer____________________ Date: _2024-01-14 ___________________________

Author: _Galarneau et al. DOI: 10.1182/blood-2013-01-478776 __Year: 2013 _Record Number: BI

	Yes	No	Unclear	Not applicable
1. Were the groups similar and recruited from the same population?	☐	☒	☐	☐
2. Were the exposures measured similarly to assign people to both exposed and unexposed groups?	☒	☐	☐	☐
3. Was the exposure measured in a valid and reliable way?	☒	☐	☐	☐
4. Were confounding factors identified?	☒	☐	☐	☐
5. Were strategies to deal with confounding factors stated?	☒	☐	☐	☐
6. Were all groups/participants free of the outcome at the start of the study (or at the moment of exposure)?	☐	☒	☐	☐
7. Were the outcomes measured in a valid and reliable way?	☒	☐	☐	☐
8. Was the follow up time reported and sufficient to be long enough for outcomes to occur?	☒	☐	☐	☐
9. Was follow up complete, and if not, were the reasons to loss to follow up described and explored?	☐	☐	☐	☒
10. Were strategies to address incomplete follow up utilized?	☐	☐	☐	☒
11. Was appropriate statistical analysis used?	☒	☐	☐	☐

Overall appraisal: Include ☒ Exclude ☐ Seek further info ☐

Comments (Including reason for exclusion)

- Cohort study with a validation cohort of 3200 SCD multi-centre patients (with 2100 HbSS patients), characterized elsewhere by the Adult Sickle Cell Clinic of Georgia Health Sciences University (GHSU) Sickle Cell Centre, and a different validation cohort of 318 adult SCD patients (20-74 yrs, F:M 169:149) and comprehensive analysis of ACS, VOC correlation with different SNPs, accounting statistically for sex, age, alpha-thalassemia and (depending on the cohort) the first 2 or 10 principal components. There was inter-group variation in age.

Critical Appraisal Checklist for Cohort Studies - © JBI, 2020. All rights reserved. JBI grants use of these tools for research purposes only. All other enquiries should be sent to jbisynthesis@adelaide.edu.au.

JBI CRITICAL APPRAISAL CHECKLIST FOR COHORT STUDIES

Reviewer: __Froso Sophocleous__________________ Date: _2024-01-12 ___________________________

Author: Sharan et al. (doi: 10.1046/j.1365-2141.2003.04762.x.) ___Year: _2004 Record Number: BJ

	Yes	No	Unclear	Not applicable
1. Were the groups similar and recruited from the same population?	☐	☐	☐	☒
2. Were the exposures measured similarly to assign people to both exposed and unexposed groups?	☐	☐	☐	☒
3. Was the exposure measured in a valid and reliable way?	☒	☐	☐	☐
4. Were confounding factors identified?	☒	☐	☐	☐
5. Were strategies to deal with confounding factors stated?	☒	☐	☐	☐
6. Were all groups/participants free of the outcome at the start of the study (or at the moment of exposure)?	☒	☐	☐	☐
7. Were the outcomes measured in a valid and reliable way?	☒	☐	☐	☐
8. Was the follow up time reported and sufficient to be long enough for outcomes to occur?	☐	☐	☐	☒
9. Was follow up complete, and if not, were the reasons to loss to follow up described and explored?	☐	☐	☐	☒
10. Were strategies to address incomplete follow up utilized?	☐	☐	☐	☒
11. Was appropriate statistical analysis used?	☒	☐	☐	☐

Overall appraisal: Include ☒ Exclude ☐ Seek further info ☐

Comments (Including reason for exclusion)

- A single cohort with no controls: 87 African–American patients with SCD enrolled from two centres.

Critical Appraisal Checklist for Cohort Studies - © JBI, 2020. All rights reserved. JBI grants use of these tools for research purposes only. All other enquiries should be sent to jbisynthesis@adelaide.edu.au.

JBI CRITICAL APPRAISAL CHECKLIST FOR CASE REPORTS

Reviewer: __Carsten W Lederer__________________ Date: _2024-12-03 __________________________

Author: _Pessoa et al. (doi: 10.1002/jha2.325)________________Year: 2021__Record Number: BL

	Yes	No	Unclear	Not applicable
1. Were patient's demographic characteristics clearly described?	☒	☐	☐	☐
2. Was the patient's history clearly described and presented as a timeline?	☒	☐	☐	☐
3. Was the current clinical condition of the patient on presentation clearly described?	☒	☐	☐	☐
4. Were diagnostic tests or assessment methods and the results clearly described?	☒	☐	☐	☐
5. Was the intervention(s) or treatment procedure(s) clearly described?	☒	☐	☐	☐
6. Was the post-intervention clinical condition clearly described?	☒	☐	☐	☐
7. Were adverse events (harms) or unanticipated events identified and described?	☒	☐	☐	☐
8. Does the case report provide takeaway lessons?	☐	☐	☒	☐

Overall appraisal: Include ☐ Exclude ☒ Seek further info ☐

Comments (Including reason for exclusion)

- Case report for three Brazilian paediatric patients with severe SCD ACS and pain complications during COVID-19 infection who held missense mutations in pattern recognition receptor pathway components required for innate immune defences against viruses, specifically the ssRNA-specific TLR7 (X-chromosomal) and the TLR4-associated TIR adaptor protein TIRAP (chromosome 11), respectively. Included only in the text, to illustrate how genetic modifiers may amplify the effect of environmental factors on the severity of the pain phenotype.

Critical Appraisal Checklist for Cohort Studies - © JBI, 2020. All rights reserved. JBI grants use of these tools for research purposes only. All other enquiries should be sent to jbisynthesis@adelaide.edu.au.

JBI CRITICAL APPRAISAL CHECKLIST FOR COHORT STUDIES

Reviewer: Carsten W Lederer Date: 2023-12-05

Author: Cancio et al. (DOI: 10.1002/pbc.26596) Year: 2017 Record Number: BO

	Yes	No	Unclear	Not applicable
1. Were the groups similar and recruited from the same population?	☐	☐	☐	☒
2. Were the exposures measured similarly to assign people to both exposed and unexposed groups?	☐	☐	☐	☒
3. Was the exposure measured in a valid and reliable way?	☒	☐	☐	☐
4. Were confounding factors identified?	☐	☐	☐	☒
5. Were strategies to deal with confounding factors stated?	☐	☐	☐	☒
6. Were all groups/participants free of the outcome at the start of the study (or at the moment of exposure)?	☐	☒	☐	☐
7. Were the outcomes measured in a valid and reliable way?	☒	☐	☐	☐
8. Was the follow up time reported and sufficient to be long enough for outcomes to occur?	☒	☐	☐	☐
9. Was follow up complete, and if not, were the reasons to loss to follow up described and explored?	☐	☐	☐	☒
10. Were strategies to address incomplete follow up utilized?	☐	☐	☐	☒
11. Was appropriate statistical analysis used?	☐	☐	☐	☒

Overall appraisal: Include ☐ Exclude ☒ Seek further info ☐

Comments (Including reason for exclusion)

- Cohort study. HbS/Black(Aγδβ)0 followed over 60.7 combined patient years for 9 patients. SCD complications VOC, dactylitis, cholelithiasis, osteonecrosis, ACS, osteomyelitis, but not splenic sequestration, stroke, sepsis or requirement for erythroid transfusion. Most comprehensive description of HbS/Black(Aγδβ)0 patients with explicit statement of VOC occurrence per patient (P1,4,6,9), severe lower extremity pain (P2,3), leg pain (P8), explicit exclusion of VOC (P5,7). **Included in the section *The influence of HBB alleles*.**

Critical Appraisal Checklist for Cohort Studies - © JBI, 2020. All rights reserved. JBI grants use of these tools for research purposes only. All other enquiries should be sent to jbisynthesis@adelaide.edu.au.

JBI CRITICAL APPRAISAL CHECKLIST FOR COHORT STUDIES

Reviewer: Carsten W Lederer Date: 2023-12-05

Author: Adekile et al. (DOI: 10.1097/MPH.0000000000000747) Year: 2017 Record Number: BP

	Yes	No	Unclear	Not applicable
1. Were the groups similar and recruited from the same population?	☒	☐	☐	☐
2. Were the exposures measured similarly to assign people to both exposed and unexposed groups?	☒	☐	☐	☐
3. Was the exposure measured in a valid and reliable way?	☒	☐	☐	☐
4. Were confounding factors identified?	☒	☐	☐	☐
5. Were strategies to deal with confounding factors stated?	☐	☒	☐	☐
6. Were all groups/participants free of the outcome at the start of the study (or at the moment of exposure)?	☐	☒	☐	☐
7. Were the outcomes measured in a valid and reliable way?	☒	☐	☐	☐
8. Was the follow up time reported and sufficient to be long enough for outcomes to occur?	☒	☐	☐	☐
9. Was follow up complete, and if not, were the reasons to loss to follow up described and explored?	☐	☐	☐	☒
10. Were strategies to address incomplete follow up utilized?	☐	☐	☐	☒
11. Was appropriate statistical analysis used?	☐	☐	☒	☐

Overall appraisal: Include ☐ Exclude ☒ Seek further info ☐

Comments (Including reason for exclusion)

- Cohort study. 102 patients (5 -24 years), 32 Sβ, 70 SS both gender-balanced. Greater heterogeneity of βS group was remarked. More frequent VOC and 'more severe pain phenotype' for Sβ vs SS, but similar frequency of ACS, gallstones, blood transfusion; Sβ were all non-transfused. No statement of normality tests and reliance on separate Student's t tests. **Included in the section *The influence of HBB alleles*.**

Critical Appraisal Checklist for Cohort Studies - © JBI, 2020. All rights reserved. JBI grants use of these tools for research purposes only. All other enquiries should be sent to jbisynthesis@adelaide.edu.au.

JBI CRITICAL APPRAISAL CHECKLIST FOR CASE REPORTS

Reviewer: __Carsten W Lederer__________________ Date: _2023-12-05 ___________________________

Author: __Goode et al. (DOI: 10.1097/MPH.0000000000001882)__Year: 2020__Record Number: BQ

	Yes	No	Unclear	Not applicable
1. Were patient's demographic characteristics clearly described?	☒	☐	☐	☐
2. Was the patient's history clearly described and presented as a timeline?	☒	☐	☐	☐
3. Was the current clinical condition of the patient on presentation clearly described?	☒	☐	☐	☐
4. Were diagnostic tests or assessment methods and the results clearly described?	☒	☐	☐	☐
5. Was the intervention(s) or treatment procedure(s) clearly described?	☒	☐	☐	☐
6. Was the post-intervention clinical condition clearly described?	☒	☐	☐	☐
7. Were adverse events (harms) or unanticipated events identified and described?	☒	☐	☐	☐
8. Does the case report provide takeaway lessons?	☒	☐	☐	☐

Overall appraisal: Include ☐ Exclude ☒ Seek further info ☐

Comments (Including reason for exclusion)

- Case report. 6-year follow-up, including VOCs, osteomyelitis, priapism and application of hydroxyurea. Inclusion as single case report for the rarity of the genotype and quality of assessment and for its underlining the message of required vigilance and application of full molecular/mass spectrometry assessment, in cases where apparent diagnosis/phenotypic discrepancies occur, in order to guide disease management. **Included in the section *The influence of HBB alleles*.**

Critical Appraisal Checklist for Cohort Studies - © JBI, 2020. All rights reserved. JBI grants use of these tools for research purposes only. All other enquiries should be sent to jbisynthesis@adelaide.edu.au.

JBI CRITICAL APPRAISAL CHECKLIST FOR COHORT STUDIES

Reviewer: Carsten W Lederer Date: 2023-12-05

Author: Calderwood et al. (DOI: 10.1097/MPH.0000000000002546) Year: 2023 Record Number: BR

	Yes	No	Unclear	Not applicable
1. Were the groups similar and recruited from the same population?	☐	☐	☐	☒
2. Were the exposures measured similarly to assign people to both exposed and unexposed groups?	☐	☐	☐	☒
3. Was the exposure measured in a valid and reliable way?	☒	☐	☐	☐
4. Were confounding factors identified?	☐	☒	☐	☐
5. Were strategies to deal with confounding factors stated?	☐	☒	☐	☐
6. Were all groups/participants free of the outcome at the start of the study (or at the moment of exposure)?	☐	☒	☐	☐
7. Were the outcomes measured in a valid and reliable way?	☒	☐	☐	☐
8. Was the follow up time reported and sufficient to be long enough for outcomes to occur?	☒	☐	☐	☐
9. Was follow up complete, and if not, were the reasons to loss to follow up described and explored?	☒	☐	☐	☐
10. Were strategies to address incomplete follow up utilized?	☐	☐	☒	☒
11. Was appropriate statistical analysis used?	☐	☐	☒	☐

Overall appraisal: Include ☐ Exclude ☒ Seek further info ☐

Comments (Including reason for exclusion)

- Combined cohort overview for 38 SS patients with COVID-19 infection, plus one case of HbSD-Los Angeles with COVID-19 infection. No meaningful description of VOC effect apparent, no focus on the genetic basis of differences. Completeness of follow-up stated above refers to COVID-19 infection, not to SCD. **Included in the section *The influence of environmental factors.***

Critical Appraisal Checklist for Cohort Studies - © JBI, 2020. All rights reserved. JBI grants use of these tools for research purposes only. All other enquiries should be sent to jbisynthesis@adelaide.edu.au.

JBI CRITICAL APPRAISAL CHECKLIST FOR COHORT STUDIES

Reviewer: __Carsten W Lederer__________________ Date: _2023-12-05 ___________________________

Author: _Rezende et al. (DOI: 10.1016/j.jped.2017.09.010)_______Year: 2018_ Record Number: BS

	Yes	No	Unclear	Not applicable
1. Were the groups similar and recruited from the same population?	☐	☐	☐	☒
2. Were the exposures measured similarly to assign people to both exposed and unexposed groups?	☐	☐	☐	☒
3. Was the exposure measured in a valid and reliable way?	☒	☐	☐	☐
4. Were confounding factors identified?	☐	☒	☐	☐
5. Were strategies to deal with confounding factors stated?	☐	☐	☐	☒
6. Were all groups/participants free of the outcome at the start of the study (or at the moment of exposure)?	☐	☒	☐	☐
7. Were the outcomes measured in a valid and reliable way?	☒	☐	☐	☐
8. Was the follow up time reported and sufficient to be long enough for outcomes to occur?	☒	☐	☐	☐
9. Was follow up complete, and if not, were the reasons to loss to follow up described and explored?	☐	☐	☐	☒
10. Were strategies to address incomplete follow up utilized?	☐	☐	☐	☒
11. Was appropriate statistical analysis used?	☐	☒	☐	☐

Overall appraisal: Include ☐ Exclude ☒ Seek further info ☐

Comments (Including reason for exclusion)

- Cohort study with 461 HbSC infants (9.2 yrs, 1-16.6), followed up for 2 to 15 years (born 1/1/1999 to 31/12/2014 follow-up to 31/12/2014). No statistical comparison with HbSS group, but statistics for VOC are presented across the study population (51 VOCs per 100 patient-years, approximately 25% of children without VOC). Observational data for VOC, infections, acute splenic sequestration crisis (ASSC), priapism, ophthalmologic, orthopaedic, cardiologic and acute cerebrovascular events. **Included in the section *The influence of HBB alleles*.**

Critical Appraisal Checklist for Cohort Studies - © JBI, 2020. All rights reserved. JBI grants use of these tools for research purposes only. All other enquiries should be sent to jbisynthesis@adelaide.edu.au.

JBI CRITICAL APPRAISAL CHECKLIST FOR COHORT STUDIES

Reviewer: Carsten W Lederer Date: 2023-12-05

Author: Jain et al. (DOI: 10.1007/s12098-016-2081-7) Year: 2016 Record Number: BT

	Yes	No	Unclear	Not applicable
1. Were the groups similar and recruited from the same population?	☒	☐	☐	☐
2. Were the exposures measured similarly to assign people to both exposed and unexposed groups?	☐	☐	☐	☒
3. Was the exposure measured in a valid and reliable way?	☒	☐	☐	☐
4. Were confounding factors identified?	☐	☒	☐	☐
5. Were strategies to deal with confounding factors stated?	☐	☐	☐	☒
6. Were all groups/participants free of the outcome at the start of the study (or at the moment of exposure)?	☐	☒	☐	☐
7. Were the outcomes measured in a valid and reliable way?	☒	☐	☐	☐
8. Was the follow up time reported and sufficient to be long enough for outcomes to occur?	☐	☐	☐	☒
9. Was follow up complete, and if not, were the reasons to loss to follow up described and explored?	☐	☐	☐	☒
10. Were strategies to address incomplete follow up utilized?	☐	☐	☐	☒
11. Was appropriate statistical analysis used?	☒	☐	☐	☐

Overall appraisal: Include ☐ Exclude ☒ Seek further info ☐

Comments (Including reason for exclusion)

- Cohort and retrospective study with HbSS (49) HbβS (36) and HbD Punjab (6) patients. No difference in dactylitis, bone pain crises, ACS, Hb, Rb count, whereas HbβS patients show higher blood transfusion requirement. Tests by χ2, Fisher's exact and Mann-Whitney U tests. β-Mutations were stratified, gender balance almost perfect for SS and βS (but 100% male for SD-Punjab). One one-week assessment, with reference to medical history. **Included in the section *The influence of HBB alleles*.**

Critical Appraisal Checklist for Cohort Studies - © JBI, 2020. All rights reserved. JBI grants use of these tools for research purposes only. All other enquiries should be sent to jbisynthesis@adelaide.edu.au.

JBI CRITICAL APPRAISAL CHECKLIST FOR COHORT STUDIES

Reviewer: Carsten W Lederer Date: 2023-12-05

Author: Adekile et al. (DOI: 10.3109/03630269.2011.617230) Year: 2011 Record Number: BU

	Yes	No	Unclear	Not applicable
1. Were the groups similar and recruited from the same population?	☒	☐	☐	☐
2. Were the exposures measured similarly to assign people to both exposed and unexposed groups?	☒	☐	☐	☐
3. Was the exposure measured in a valid and reliable way?	☒	☐	☐	☐
4. Were confounding factors identified?	☐	☐	☒	☐
5. Were strategies to deal with confounding factors stated?	☐	☐	☒	☐
6. Were all groups/participants free of the outcome at the start of the study (or at the moment of exposure)?	☐	☒	☐	☐
7. Were the outcomes measured in a valid and reliable way?	☒	☐	☐	☐
8. Was the follow up time reported and sufficient to be long enough for outcomes to occur?	☐	☐	☐	☒
9. Was follow up complete, and if not, were the reasons to loss to follow up described and explored?	☐	☐	☐	☒
10. Were strategies to address incomplete follow up utilized?	☐	☐	☐	☒
11. Was appropriate statistical analysis used?	☐	☐	☒	☐

Overall appraisal: Include ☐ Exclude ☒ Seek further info ☐

Comments (Including reason for exclusion)

- Cohort study, where **merely summary statistics are apparent from tables**, though the text also talks about significant findings between groups, but without detailing the method. VOC assessed semi-quantitatively in a subgroup of patients.

Critical Appraisal Checklist for Cohort Studies - © JBI, 2020. All rights reserved. JBI grants use of these tools for research purposes only. All other enquiries should be sent to jbisynthesis@adelaide.edu.au.

JBI CRITICAL APPRAISAL CHECKLIST FOR COHORT STUDIES

Reviewer: Carsten W Lederer Date: 2023-12-05

Author: Zimmerman et al. [DOI: 10.1002/(sici)1096-8652(199904)60:4<279::aid-ajh5>3.0.co;2-2] Year: 1999 Record Number: BV

	Yes	No	Unclear	Not applicable
1. Were the groups similar and recruited from the same population?	☐	☐	☐	☒
2. Were the exposures measured similarly to assign people to both exposed and unexposed groups?	☐	☐	☐	☒
3. Was the exposure measured in a valid and reliable way?	☒	☐	☐	☐
4. Were confounding factors identified?	☐	☐	☐	☒
5. Were strategies to deal with confounding factors stated?	☐	☐	☐	☒
6. Were all groups/participants free of the outcome at the start of the study (or at the moment of exposure)?	☐	☒	☐	☐
7. Were the outcomes measured in a valid and reliable way?	☒	☐	☐	☐
8. Was the follow up time reported and sufficient to be long enough for outcomes to occur?	☒	☐	☐	☐
9. Was follow up complete, and if not, were the reasons to loss to follow up described and explored?	☐	☐	☐	☒
10. Were strategies to address incomplete follow up utilized?	☐	☐	☐	☒
11. Was appropriate statistical analysis used?	☐	☐	☐	☒

Overall appraisal: Include ☐ Exclude ☒ Seek further info ☐

Comments (Including reason for exclusion)

- Cohort study enumeration of VOC, ACS and other events; no statistical comparison to other disease groups. Details are given for all 13 patients and a list of clinical events, including ACS (11/13, including one death), VOC (9/13), dactylitis (7/13), childhood deaths by infection (2/13), adult death by multiorgan failure (1/13) and anecdotal comparison with (more haemolytic) HbSS and (milder, with higher gelling point and higher oxygen affinity) HbSC. **Included in the section *The influence of HBB alleles*.**

Critical Appraisal Checklist for Cohort Studies - © JBI, 2020. All rights reserved. JBI grants use of these tools for research purposes only. All other enquiries should be sent to jbisynthesis@adelaide.edu.au.

JBI CRITICAL APPRAISAL CHECKLIST FOR COHORT STUDIES

Reviewer: Carsten W Lederer Date: 2023-12-05

Author: Torres et al. (DOI: 10.1080/03630269.2016.1222295) Year: 2016 Record Number: BW

	Yes	No	Unclear	Not applicable
1. Were the groups similar and recruited from the same population?	☐	☐	☐	☒
2. Were the exposures measured similarly to assign people to both exposed and unexposed groups?	☐	☐	☐	☒
3. Was the exposure measured in a valid and reliable way?	☒	☐	☐	☐
4. Were confounding factors identified?	☐	☐	☐	☒
5. Were strategies to deal with confounding factors stated?	☐	☐	☐	☒
6. Were all groups/participants free of the outcome at the start of the study (or at the moment of exposure)?	☐	☒	☐	☐
7. Were the outcomes measured in a valid and reliable way?	☒	☐	☐	☐
8. Was the follow up time reported and sufficient to be long enough for outcomes to occur?	☒	☐	☐	☐
9. Was follow up complete, and if not, were the reasons to loss to follow up described and explored?	☐	☐	☐	☒
10. Were strategies to address incomplete follow up utilized?	☐	☐	☐	☒
11. Was appropriate statistical analysis used?	☐	☒	☐	☐

Overall appraisal: Include ☐ Exclude ☒ Seek further info ☐

Comments (Including reason for exclusion)

- Cohort study, assessing 12 patients of S/Hb D-Punjab genotype and the Bantu [– + – – – –] haplotype, chosen for absence of HU treatment, without statistical comparison with other disease groups and with enumeration of recurrent VOC (8/12), sporadic VOC (5/12; with inadvertent double-counting of patient SD03 for both VOC enumerations), ACS (2/12), absence of symptoms (3/12) and occurrence of wider symptoms in two patients (2/12), including of priapism and persistent leg ulcer in one and avascular necrosis and bone infarction in another. **Included in the section *The influence of HBB alleles*.**

Critical Appraisal Checklist for Cohort Studies - © JBI, 2020. All rights reserved. JBI grants use of these tools for research purposes only. All other enquiries should be sent to jbisynthesis@adelaide.edu.au.

JBI CRITICAL APPRAISAL CHECKLIST FOR COHORT STUDIES

Reviewer: Carsten W Lederer Date: 2024-01-14

Author: Bean et al. (DOI: 10.1111/bjh.12507) Year: 2013 Record Number: BX

	Yes	No	Unclear	Not applicable
1. Were the groups similar and recruited from the same population?	☐	☐	☐	☒
2. Were the exposures measured similarly to assign people to both exposed and unexposed groups?	☐	☐	☐	☒
3. Was the exposure measured in a valid and reliable way?	☒	☐	☐	☐
4. Were confounding factors identified?	☒	☐	☐	☐
5. Were strategies to deal with confounding factors stated?	☒	☐	☐	☐
6. Were all groups/participants free of the outcome at the start of the study (or at the moment of exposure)?	☐	☒	☐	☐
7. Were the outcomes measured in a valid and reliable way?	☒	☐	☐	☐
8. Was the follow up time reported and sufficient to be long enough for outcomes to occur?	☒	☐	☐	☐
9. Was follow up complete, and if not, were the reasons to loss to follow up described and explored?	☒	☐	☐	☐
10. Were strategies to address incomplete follow up utilized?	☐	☐	☐	☒
11. Was appropriate statistical analysis used?	☒	☐	☐	☐

Overall appraisal: Include ☒ Exclude ☐ Seek further info ☐

Comments (Including reason for exclusion)

- Cohort study for SCD, excluding first-degree relatives and patients with HU treatment, without VOC and ACS data and with HbSβ0 genotype, to focus on 820 paediatric HbSS (SCA) patients, showing statistical association of HBB (SNP-based) haplotypes with HbF levels and ACS, but not with VOC and silent cerebral infarction. HBB haplotype and HMOX1 promoter genotype were assessed for the whole cohort; 63 (7.7%), carrying rare haplotypes of <1% frequency, and 24 (2.9%), H2/H3-heterozygous and thus HBB-haplotype-ambiguous individuals, were excluded. **Included in text and table for β haplotype statistics.**

Critical Appraisal Checklist for Cohort Studies - © JBI, 2020. All rights reserved. JBI grants use of these tools for research purposes only. All other enquiries should be sent to jbisynthesis@adelaide.edu.au.

JBI CRITICAL APPRAISAL CHECKLIST FOR COHORT STUDIES

Reviewer: Carsten W Lederer Date: 2024-01-14

Author: Powars et al. (DOI: 10.1097/00043426-199023000-00022) Year: 1990 Record Number: BK/BY

	Yes	No	Unclear	Not applicable
1. Were the groups similar and recruited from the same population?	X			
2. Were the exposures measured similarly to assign people to both exposed and unexposed groups?	X			
3. Was the exposure measured in a valid and reliable way?	X			
4. Were confounding factors identified?			X	
5. Were strategies to deal with confounding factors stated?			X	
6. Were all groups/participants free of the outcome at the start of the study (or at the moment of exposure)?		X		
7. Were the outcomes measured in a valid and reliable way?	X			
8. Was the follow up time reported and sufficient to be long enough for outcomes to occur?	X			
9. Was follow up complete, and if not, were the reasons to loss to follow up described and explored?				X
10. Were strategies to address incomplete follow up utilized?				X
11. Was appropriate statistical analysis used?			X	

Overall appraisal: Include [X] Exclude [ ] Seek further info [ ]

Comments (Including reason for exclusion)

- Cohort study based on 221 HbSS patients characterized in earlier studies, with varying HBB haplotypes and unspecified alpha-thalassemia genotypes. Tests applied for group-wise comparison (including whether parametric/non-parametric or corrected for multiple comparisons) are unclear. Apparent significance of HBB haplotypes for bone infarct (10^{-5}), VOC precipitating illness (P=0.002), hospital admissions (0.002), priapism (0.025) and fractures (0.038) across mild Senegal/Benin and Benin/Benin, CAR (Central African Republic)/Benin haplotypes.

Critical Appraisal Checklist for Cohort Studies - © JBI, 2020. All rights reserved. JBI grants use of these tools for research purposes only. All other enquiries should be sent to jbisynthesis@adelaide.edu.au.

JBI CRITICAL APPRAISAL CHECKLIST FOR CASE CONTROL STUDIES

Reviewer: Carsten W Lederer Date: 2024-01-14

Author: Adekile & Haider (DOI: 10.1159/000320302) Year: 2010 Record Number: BZ/BM

	Yes	No	Unclear	Not applicable
1. Were the groups comparable other than the presence of disease in cases or the absence of disease in controls?	☐	☒	☐	☐
2. Were cases and controls matched appropriately?	☒	☐	☐	☐
3. Were the same criteria used for identification of cases and controls?	☒	☐	☐	☐
4. Was exposure measured in a standard, valid and reliable way?	☒	☐	☐	☐
5. Was exposure measured in the same way for cases and controls?	☒	☐	☐	☐
6. Were confounding factors identified?	☐	☒	☐	☐
7. Were strategies to deal with confounding factors stated?	☐	☒	☐	☐
8. Were outcomes assessed in a standard, valid and reliable way for cases and controls?	☒	☐	☐	☐
9. Was the exposure period of interest long enough to be meaningful?	☒	☐	☐	☐
10. Was appropriate statistical analysis used?	☐	☐	☒	☐

Overall appraisal: Include ☐ Exclude ☒ Seek further info ☐

Comments (Including reason for exclusion)

- Case control study with two case-control pairings, comparing 82 Kuwaiti SCD patients with 49 Kuwaiti controls (HbSS and Arab/India haplotype, only), as well as 54 Nigerian SCD patients with 32 Nigerian controls (HbSS and Benin haplotype, only). NO stratification of Nigerian cohort for VOC frequency; in the absence of statistical analysis only anecdotal value of the study. **Included in the text as an example of attempted association of β-globin haplotypes with phenotypes**.

Critical Appraisal Checklist for Cohort Studies - © JBI, 2020. All rights reserved. JBI grants use of these tools for research purposes only. All other enquiries should be sent to jbisynthesis@adelaide.edu.au.

JBI CRITICAL APPRAISAL CHECKLIST FOR COHORT STUDIES

Reviewer: __Carsten W Lederer____________________ Date: _2024-06-02 ____________________________

Author: Sheehan et al. (DOI: 10.1002/ajh.23457)_______________ Year: 2013_ Record Number: CI

	Yes	No	Unclear	Not applicable
1. Were the groups similar and recruited from the same population?	☒	☐	☐	☐
2. Were the exposures measured similarly to assign people to both exposed and unexposed groups?	☒	☐	☐	☐
3. Was the exposure measured in a valid and reliable way?	☒	☐	☐	☐
4. Were confounding factors identified?	☒	☐	☐	☐
5. Were strategies to deal with confounding factors stated?	☒	☐	☐	☐
6. Were all groups/participants free of the outcome at the start of the study (or at the moment of exposure)?	☒	☐	☐	☐
7. Were the outcomes measured in a valid and reliable way?	☒	☐	☐	☐
8. Was the follow up time reported and sufficient to be long enough for outcomes to occur?	☐	☐	☒	☐
9. Was follow up complete, and if not, were the reasons to loss to follow up described and explored?	☒	☐	☐	☐
10. Were strategies to address incomplete follow up utilized?	☐	☐	☒	☐
11. Was appropriate statistical analysis used?	☒	☐	☐	☐

Overall appraisal: Include ☒ Exclude ☐ Seek further info ☐

Comments (Including reason for exclusion)

- Cohort study across 13 institutions of 190 paediatric patients (9 to 18 months of age, mean 14 months), randomized for hydroxyurea (n=94) and placebo (n=96) treatment, with consideration of α-thalassemia trait, β-globin locus haplotype, G6PD and key SNVs (XmnI, BLC11A ×3, HBS1L-MYB ×3), sex (43% male vs 57% female) as well as time (with comparison of baseline to post-treatment [placebo or hydroxyurea] measurement]) as confounding factors/parameters of analysis.

Critical Appraisal Checklist for Cohort Studies - © JBI, 2020. All rights reserved. JBI grants use of these tools for research purposes only. All other enquiries should be sent to jbisynthesis@adelaide.edu.au.

JBI CRITICAL APPRAISAL CHECKLIST FOR COHORT STUDIES

Reviewer: __Carsten W Lederer__________________ Date: _2024-06-02 __________________________

Author: Steinberg et al. _DOI n/a (PMID: 3345344)_____________ Year: 1988_ Record Number: CJ

	Yes	No	Unclear	Not applicable
1. Were the groups similar and recruited from the same population?	☒	☐	☐	☐
2. Were the exposures measured similarly to assign people to both exposed and unexposed groups?	☒	☐	☐	☐
3. Was the exposure measured in a valid and reliable way?	☐	☐	☒	☐
4. Were confounding factors identified?	☒	☐	☐	☐
5. Were strategies to deal with confounding factors stated?	☒	☐	☐	☐
6. Were all groups/participants free of the outcome at the start of the study (or at the moment of exposure)?	☐	☒	☐	☐
7. Were the outcomes measured in a valid and reliable way?	☒	☐	☐	☐
8. Was the follow up time reported and sufficient to be long enough for outcomes to occur?	☐	☐	☐	☒
9. Was follow up complete, and if not, were the reasons to loss to follow up described and explored?	☐	☐	☐	☒
10. Were strategies to address incomplete follow up utilized?	☐	☐	☐	☒
11. Was appropriate statistical analysis used?	☒	☐	☐	☐

Overall appraisal: Include ☐ Exclude ☒ Seek further info ☐

Comments (Including reason for exclusion)

- Cohort study of 801 male HbSS patients assessed for G6PD and hexokinase deficiency and taking account of age-dependence of expression. “Genotype” detection was based on cytochemical or enzymatic assays, only. No significant correlations were detected for clinical parameters under study here, such as painful episodes, VOEs or sepsis.

Critical Appraisal Checklist for Cohort Studies - © JBI, 2020. All rights reserved. JBI grants use of these tools for research purposes only. All other enquiries should be sent to jbisynthesis@adelaide.edu.au.

JBI CRITICAL APPRAISAL CHECKLIST FOR COHORT STUDIES

Reviewer: __Carsten W Lederer__________________ Date: _2024-06-02 __________________________

Author: Benkerrou et al. (DOI: 10.1111/bjh.12590)_____________ Year: 2013_ Record Number: CK

	Yes	No	Unclear	Not applicable
1. Were the groups similar and recruited from the same population?	☒	☐	☐	☐
2. Were the exposures measured similarly to assign people to both exposed and unexposed groups?	☒	☐	☐	☐
3. Was the exposure measured in a valid and reliable way?	☒	☐	☐	☐
4. Were confounding factors identified?	☒	☐	☐	☐
5. Were strategies to deal with confounding factors stated?	☒	☐	☐	☐
6. Were all groups/participants free of the outcome at the start of the study (or at the moment of exposure)?	☐	☒	☐	☐
7. Were the outcomes measured in a valid and reliable way?	☒	☐	☐	☐
8. Was the follow up time reported and sufficient to be long enough for outcomes to occur?	☒	☐	☐	☐
9. Was follow up complete, and if not, were the reasons to loss to follow up described and explored?	☒	☐	☐	☐
10. Were strategies to address incomplete follow up utilized?	☐	☐	☐	☒
11. Was appropriate statistical analysis used?	☒	☐	☐	☐

Overall appraisal: Include ☐ Exclude ☒ Seek further info ☐

Comments (Including reason for exclusion)

- Cohort study of 437 SCD (SS, SC, Sβ-thal) neonates for a 14-year time period (1991-2005). Only male SCA patients with known G6PD status and with follow-up of at least two years and up to the age of 42 months were included. No significant correlations were detected for clinical parameters under study here, such as painful episodes, VOEs or sepsis. **Included in the text for reference to cholelithiasis.**

Critical Appraisal Checklist for Cohort Studies - © JBI, 2020. All rights reserved. JBI grants use of these tools for research purposes only. All other enquiries should be sent to jbisynthesis@adelaide.edu.au.

JBI CRITICAL APPRAISAL CHECKLIST FOR CASE CONTROL STUDIES

Reviewer: Carsten W Lederer Date: 2024-06-02

Author: Tantawy et al. (DOI: 10.1002/pbc.25234) Year: 2015 Record Number: CL

	Yes	No	Unclear	Not applicable
1. Were the groups comparable other than the presence of disease in cases or the absence of disease in controls?	☒	☐	☐	☐
2. Were cases and controls matched appropriately?	☒	☐	☐	☐
3. Were the same criteria used for identification of cases and controls?	☒	☐	☐	☐
4. Was exposure measured in a standard, valid and reliable way?	☒	☐	☐	☐
5. Was exposure measured in the same way for cases and controls?	☒	☐	☐	☐
6. Were confounding factors identified?	☒	☐	☐	☐
7. Were strategies to deal with confounding factors stated?	☒	☐	☐	☐
8. Were outcomes assessed in a standard, valid and reliable way for cases and controls?	☒	☐	☐	☐
9. Was the exposure period of interest long enough to be meaningful?	☒	☐	☐	☐
10. Was appropriate statistical analysis used?	☒	☐	☐	☐

Overall appraisal: Include ☒ Exclude ☐ Seek further info ☐

Comments (Including reason for exclusion)

- Cross-sectional case-control study of 51 SCD patients (29 male, 22 female) 10.9±3.7 years and 55 age-matched control subjects (32 male, 23 male) 11.3±3.5. Sub-analysis of SCD patients for ecNOS4 short intronic repeat allele (ecNOS4a) is informative.

Critical Appraisal Checklist for Cohort Studies - © JBI, 2020. All rights reserved. JBI grants use of these tools for research purposes only. All other enquiries should be sent to jbisynthesis@adelaide.edu.au.

JBI CRITICAL APPRAISAL CHECKLIST FOR CASE CONTROL STUDIES

Reviewer: __Carsten W Lederer__________________ Date: _2024-06-02 ___________________________

Author: Nishank et al. (DOI: 10.1038/jhg.2013.99)______________ Year: 2013_ Record Number: CM

	Yes	No	Unclear	Not applicable
1. Were the groups comparable other than the presence of disease in cases or the absence of disease in controls?	☒	☐	☐	☐
2. Were cases and controls matched appropriately?	☒	☐	☐	☐
3. Were the same criteria used for identification of cases and controls?	☒	☐	☐	☐
4. Was exposure measured in a standard, valid and reliable way?	☒	☐	☐	☐
5. Was exposure measured in the same way for cases and controls?	☒	☐	☐	☐
6. Were confounding factors identified?	☒	☐	☐	☐
7. Were strategies to deal with confounding factors stated?	☒	☐	☐	☐
8. Were outcomes assessed in a standard, valid and reliable way for cases and controls?	☐	☐	☒	☐
9. Was the exposure period of interest long enough to be meaningful?	☒	☐	☐	☐
10. Was appropriate statistical analysis used?	☒	☐	☐	☐

Overall appraisal: Include ☐ Exclude ☒ Seek further info ☐

Comments (Including reason for exclusion)

- A case control study of 150 SCD patients with age- and ethnicity-matched control subjects. SCD patients were further separated into mild (Severity score [SI])≤6.0; n=32) and severe (SI≥6.1; n=118; HbS/βthal n=5; HbSS n=113). The study of three major eNOS polymorphisms conflates chronic pain, painful crises, aseptic necrosis and priapism with many other disease aspects not of relevance for this review, as represented by a severity index (PMID: 1281601), where the combination of those relevant parameters can reach up to a score of 15 out of a maximum total of 53.

Critical Appraisal Checklist for Cohort Studies - © JBI, 2020. All rights reserved. JBI grants use of these tools for research purposes only. All other enquiries should be sent to jbisynthesis@adelaide.edu.au.

JBI CRITICAL APPRAISAL CHECKLIST FOR CASE CONTROL STUDIES

Reviewer: Carsten W Lederer Date: 2024-06-02

Author: Yousry et al. (DOI: 10.1080/10245332.2016.1142710) Year: 2016 Record Number: CN

	Yes	No	Unclear	Not applicable
1. Were the groups comparable other than the presence of disease in cases or the absence of disease in controls?	☒	☐	☐	☐
2. Were cases and controls matched appropriately?	☒	☐	☐	☐
3. Were the same criteria used for identification of cases and controls?	☒	☐	☐	☐
4. Was exposure measured in a standard, valid and reliable way?	☒	☐	☐	☐
5. Was exposure measured in the same way for cases and controls?	☒	☐	☐	☐
6. Were confounding factors identified?	☒	☐	☐	☐
7. Were strategies to deal with confounding factors stated?	☒	☐	☐	☐
8. Were outcomes assessed in a standard, valid and reliable way for cases and controls?	☒	☐	☐	☐
9. Was the exposure period of interest long enough to be meaningful?	☒	☐	☐	☐
10. Was appropriate statistical analysis used?	☐	☐	☒	☐

Overall appraisal: Include ☐ Exclude ☒ Seek further info ☐

Comments (Including reason for exclusion)

- An Egyptian case control study of 100 SCD vs 80 control subjects and similar age and sex distribution in both groups, excluding subjects under 18 yrs or with unrelated comorbidities. 2.5-year follow-up measured VOC, ACS, renal injury and pulmonary hypertension for correlation with two NOS3 polymorphisms. Apparently p values were not adjusted for multiple testing correction and might individually over-estimate the apparent significance. **Included only in the text**, owing to those concerns.

Critical Appraisal Checklist for Cohort Studies - © JBI, 2020. All rights reserved. JBI grants use of these tools for research purposes only. All other enquiries should be sent to jbisynthesis@adelaide.edu.au.

JBI CRITICAL APPRAISAL CHECKLIST FOR COHORT STUDIES

Reviewer: Carsten W Lederer Date: 2024-06-02

Author: Oliveira et al. (DOI: 10.1159/000220335) Year: 2009 Record Number: CO

	Yes	No	Unclear	Not applicable
1. Were the groups similar and recruited from the same population?	X			
2. Were the exposures measured similarly to assign people to both exposed and unexposed groups?	X			
3. Was the exposure measured in a valid and reliable way?	X			
4. Were confounding factors identified?	X			
5. Were strategies to deal with confounding factors stated?	X			
6. Were all groups/participants free of the outcome at the start of the study (or at the moment of exposure)?		X		
7. Were the outcomes measured in a valid and reliable way?	X			
8. Was the follow up time reported and sufficient to be long enough for outcomes to occur?	X			
9. Was follow up complete, and if not, were the reasons to loss to follow up described and explored?	X			
10. Were strategies to address incomplete follow up utilized?				X
11. Was appropriate statistical analysis used?	X			

Overall appraisal: Include [X] Exclude [ ] Seek further info [ ]

Comments (Including reason for exclusion)

- A cohort study of 422 symptomatic Brazilian SCA patients aged 0-71 years (47.3% male, 52.7% female). 87 children aged 0 to 5 years (median: 2.5 years, 48.3% male, 51.7% female) were analysed for MBL2 polymorphism association with VOC and RTI as a homogeneous subgroup that had been uniformly vaccinated and were given prophylactic penicillin. Age medians and ranges per subgroups are not given.

Critical Appraisal Checklist for Cohort Studies - © JBI, 2020. All rights reserved. JBI grants use of these tools for research purposes only. All other enquiries should be sent to jbisynthesis@adelaide.edu.au.

JBI CRITICAL APPRAISAL CHECKLIST FOR COHORT STUDIES

Reviewer: __Carsten W Lederer__________________ Date: _2024-06-02 __________________________

Author: Mendonça _et al. (DOI: 10.1016/j.bcmd.2010.02.004)____Year: 2010__Record Number: CP

	Yes	No	Unclear	Not applicable
1. Were the groups similar and recruited from the same population?	☒	☐	☐	☐
2. Were the exposures measured similarly to assign people to both exposed and unexposed groups?	☒	☐	☐	☐
3. Was the exposure measured in a valid and reliable way?	☒	☐	☐	☐
4. Were confounding factors identified?	☒	☐	☐	☐
5. Were strategies to deal with confounding factors stated?	☒	☐	☐	☐
6. Were all groups/participants free of the outcome at the start of the study (or at the moment of exposure)?	☐	☒	☐	☐
7. Were the outcomes measured in a valid and reliable way?	☒	☐	☐	☐
8. Was the follow up time reported and sufficient to be long enough for outcomes to occur?	☒	☐	☐	☐
9. Was follow up complete, and if not, were the reasons to loss to follow up described and explored?	☒	☐	☐	☐
10. Were strategies to address incomplete follow up utilized?	☐	☐	☐	☒
11. Was appropriate statistical analysis used?	☒	☐	☐	☐

Overall appraisal: Include ☒ Exclude ☐ Seek further info ☐

Comments (Including reason for exclusion)

- A cohort study 87 children one year after the study #CO Oliveira et al, 2009 i.e. with children aged 1 to 6 years (3.46±1.49) analysed a combination of 2 loci as combined haplotypes for MBL2 (promoter region -221 XY SNV and 3 ORF SNVs of the so-called A/O haplotype) as to their effect on VOC frequency. VOC groups were (unusually) separated by the frequency of VOC episodes divided by the proband age at the end of the study.

Critical Appraisal Checklist for Cohort Studies - © JBI, 2020. All rights reserved. JBI grants use of these tools for research purposes only. All other enquiries should be sent to jbisynthesis@adelaide.edu.au.

JBI CRITICAL APPRAISAL CHECKLIST FOR CASE CONTROL STUDIES

Reviewer: _ _Carsten W Lederer____________________ Date: _2024-06-03 ______________________________

Author: Redha et al. (DOI: 10.1002/pbc.25158)__________________ Year: 2014 _Record Number: CQ

	Yes	No	Unclear	Not applicable
1. Were the groups comparable other than the presence of disease in cases or the absence of disease in controls?	☒	☐	☐	☐
2. Were cases and controls matched appropriately?	☒	☐	☐	☐
3. Were the same criteria used for identification of cases and controls?	☒	☐	☐	☐
4. Was exposure measured in a standard, valid and reliable way?	☒	☐	☐	☐
5. Was exposure measured in the same way for cases and controls?	☒	☐	☐	☐
6. Were confounding factors identified?	☒	☐	☐	☐
7. Were strategies to deal with confounding factors stated?	☒	☐	☐	☐
8. Were outcomes assessed in a standard, valid and reliable way for cases and controls?	☒	☐	☐	☐
9. Was the exposure period of interest long enough to be meaningful?	☒	☐	☐	☐
10. Was appropriate statistical analysis used?	☒	☐	☐	☐

Overall appraisal: Include ☒ Exclude ☐ Seek further info ☐

Comments (Including reason for exclusion)

- A retrospective case-control study with 351 SCA subjects based on hemoglobin profile, investigating ACS (n=90, 46 male, 43 female [sic]) vs non-ACS (n=261, 156 male, 110 female [sic]) subjects for association with VEGFA rs3025020.

Critical Appraisal Checklist for Cohort Studies - © JBI, 2020. All rights reserved. JBI grants use of these tools for research purposes only. All other enquiries should be sent to jbisynthesis@adelaide.edu.au.

JBI CRITICAL APPRAISAL CHECKLIST FOR COHORT STUDIES

Reviewer: Carsten W Lederer Date: 2024-06-03

Author: Ashley-Koch et al. (DOI: 10.1111/j.1365-2141.2011.08832.x) Year: 2011 Record Number: CR

	Yes	No	Unclear	Not applicable
1. Were the groups similar and recruited from the same population?	☒	☐	☐	☐
2. Were the exposures measured similarly to assign people to both exposed and unexposed groups?	☒	☐	☐	☐
3. Was the exposure measured in a valid and reliable way?	☒	☐	☐	☐
4. Were confounding factors identified?	☒	☐	☐	☐
5. Were strategies to deal with confounding factors stated?	☒	☐	☐	☐
6. Were all groups/participants free of the outcome at the start of the study (or at the moment of exposure)?	☒	☐	☐	☐
7. Were the outcomes measured in a valid and reliable way?	☒	☐	☐	☐
8. Was the follow up time reported and sufficient to be long enough for outcomes to occur?	☒	☐	☐	☐
9. Was follow up complete, and if not, were the reasons to loss to follow up described and explored?	☒	☐	☐	☐
10. Were strategies to address incomplete follow up utilized?	☐	☐	☐	☒
11. Was appropriate statistical analysis used?	☒	☐	☐	☐

Overall appraisal: Include ☐ Exclude ☒ Seek further info ☐

Comments (Including reason for exclusion)

- Cohort study of 520 SCD patients of different HBB genotypes and ethnic origin. With focus on proteinuria as a proxy for early nephropathy, **this study did not touch on any disease aspects of relevance for this review**.

Critical Appraisal Checklist for Cohort Studies - © JBI, 2020. All rights reserved. JBI grants use of these tools for research purposes only. All other enquiries should be sent to jbisynthesis@adelaide.edu.au.

JBI CRITICAL APPRAISAL CHECKLIST FOR CASE CONTROL STUDIES

Reviewer: Carsten W Lederer Date: 2024-06-03

Author: Baldwin et al. (DOI: 10.1182/blood-2005-02-0548) Year: 2005 Record Number: CS

	Yes	No	Unclear	Not applicable
1. Were the groups comparable other than the presence of disease in cases or the absence of disease in controls?	☒	☐	☐	☐
2. Were cases and controls matched appropriately?	☐	☐	☒	☐
3. Were the same criteria used for identification of cases and controls?	☒	☐	☐	☐
4. Was exposure measured in a standard, valid and reliable way?	☒	☐	☐	☐
5. Was exposure measured in the same way for cases and controls?	☒	☐	☐	☐
6. Were confounding factors identified?	☒	☐	☐	☐
7. Were strategies to deal with confounding factors stated?	☒	☐	☐	☐
8. Were outcomes assessed in a standard, valid and reliable way for cases and controls?	☒	☐	☐	☐
9. Was the exposure period of interest long enough to be meaningful?	☒	☐	☐	☐
10. Was appropriate statistical analysis used?	☒	☐	☐	☐

Overall appraisal: Include ☒ Exclude ☐ Seek further info ☐

Comments (Including reason for exclusion)

- A case-control US study based on the African-American SCD cohort from the Cooperative Study of Sickle Cell Disease (CSSCD), with first report of recruitment in 1987 for 3200 patients, from infants to patients over 50 years of age and including 2100 SCA patients. The study investigated association of polymorphisms linked to bone development in connection with osteonecrosis. 442 subjects vs 455 controls were investigated by group-wise comparisons, where osteonecrosis-negative patients of ≥20 yrs were compared to patients with radiologically confirmed osteonecrosis. Most confounding factors were addressed, but the control, osteonecrosis-positive population was six years younger than controls, and no average ages were stated.

Critical Appraisal Checklist for Cohort Studies - © JBI, 2020. All rights reserved. JBI grants use of these tools for research purposes only. All other enquiries should be sent to jbisynthesis@adelaide.edu.au.

JBI CRITICAL APPRAISAL CHECKLIST FOR COHORT STUDIES

Reviewer: Carsten W Lederer Date: 2024-06-03

Author: Aguiar et al. (DOI: 10.3233/CH-168008) Year: 2016 Record Number: CT

	Yes	No	Unclear	Not applicable
1. Were the groups similar and recruited from the same population?	☒	☐	☐	☐
2. Were the exposures measured similarly to assign people to both exposed and unexposed groups?	☒	☐	☐	☐
3. Was the exposure measured in a valid and reliable way?	☒	☐	☐	☐
4. Were confounding factors identified?	☒	☐	☐	☐
5. Were strategies to deal with confounding factors stated?	☒	☐	☐	☐
6. Were all groups/participants free of the outcome at the start of the study (or at the moment of exposure)?	☐	☒	☐	☐
7. Were the outcomes measured in a valid and reliable way?	☒	☐	☐	☐
8. Was the follow up time reported and sufficient to be long enough for outcomes to occur?	☒	☐	☐	☐
9. Was follow up complete, and if not, were the reasons to loss to follow up described and explored?	☒	☐	☐	☐
10. Were strategies to address incomplete follow up utilized?	☐	☐	☐	☒
11. Was appropriate statistical analysis used?	☐	☐	☒	☐

Overall appraisal: Include ☐ Exclude ☒ Seek further info ☐

Comments (Including reason for exclusion)

- Cohort study of 26 SCA patients (18 male, 8 female) of mostly Portuguese origin. Despite focus on NOS3 variants with known association with SCD severity, **the low-powered study does not directly address any of the disease aspects of relevance for this review**. It is unclear how p-values were derived for individual parameters based on the groupwise tests stated, as no post hoc tests are indicated.

Critical Appraisal Checklist for Cohort Studies - © JBI, 2020. All rights reserved. JBI grants use of these tools for research purposes only. All other enquiries should be sent to jbisynthesis@adelaide.edu.au.

JBI CRITICAL APPRAISAL CHECKLIST FOR COHORT STUDIES

Reviewer: __Carsten W Lederer__________________ Date: _2024-06-03 ___________________________

Author: Pincez et al. (DOI: 10.3324/haematol.2022.281180)_____Year: 2023_ Record Number: CU

	Yes	No	Unclear	Not applicable
1. Were the groups similar and recruited from the same population?	☒	☐	☐	☐
2. Were the exposures measured similarly to assign people to both exposed and unexposed groups?	☒	☐	☐	☐
3. Was the exposure measured in a valid and reliable way?	☒	☐	☐	☐
4. Were confounding factors identified?	☒	☐	☐	☐
5. Were strategies to deal with confounding factors stated?	☒	☐	☐	☐
6. Were all groups/participants free of the outcome at the start of the study (or at the moment of exposure)?	☐	☐	☒	☐
7. Were the outcomes measured in a valid and reliable way?	☒	☐	☐	☐
8. Was the follow up time reported and sufficient to be long enough for outcomes to occur?	☒	☐	☐	☐
9. Was follow up complete, and if not, were the reasons to loss to follow up described and explored?	☒	☐	☐	☐
10. Were strategies to address incomplete follow up utilized?	☐	☐	☐	☒
11. Was appropriate statistical analysis used?	☒	☐	☐	☐

Overall appraisal: Include ☒ Exclude ☐ Seek further info ☐

Comments (Including reason for exclusion)

- A US study based on the African-American SCD cohort from the Cooperative Study of Sickle Cell Disease (CSSCD). The cohort study investigated association of an HbF-related polygenic trait score combining six SNPs at three loci (BCL11A [rs1427407, rs7606173], HBSL1-MYB [rs6940878, rs9389269, rs114398597], HBBP1 [rs10128556]) for analysis analysed in 1271 genotyped patients. No average ages were stated or sex ratios were stated but likely considered in the models employed.

Critical Appraisal Checklist for Cohort Studies - © JBI, 2020. All rights reserved. JBI grants use of these tools for research purposes only. All other enquiries should be sent to jbisynthesis@adelaide.edu.au.

JBI CRITICAL APPRAISAL CHECKLIST FOR COHORT STUDIES

Reviewer: __Carsten W Lederer__________________ Date: _2024-06-03 __________________________

Author: Sales et al. (DOI: 10.1007/s00277-020-04079-2) Year: 2020 Record Number: CV

	Yes	No	Unclear	Not applicable
1. Were the groups similar and recruited from the same population?	☒	☐	☐	☐
2. Were the exposures measured similarly to assign people to both exposed and unexposed groups?	☒	☐	☐	☐
3. Was the exposure measured in a valid and reliable way?	☒	☐	☐	☐
4. Were confounding factors identified?	☒	☐	☐	☐
5. Were strategies to deal with confounding factors stated?	☒	☐	☐	☐
6. Were all groups/participants free of the outcome at the start of the study (or at the moment of exposure)?	☐	☐	☒	☐
7. Were the outcomes measured in a valid and reliable way?	☒	☐	☐	☐
8. Was the follow up time reported and sufficient to be long enough for outcomes to occur?	☒	☐	☐	☐
9. Was follow up complete, and if not, were the reasons to loss to follow up described and explored?	☒	☐	☐	☐
10. Were strategies to address incomplete follow up utilized?	☐	☐	☐	☒
11. Was appropriate statistical analysis used?	☒	☐	☐	☐

Overall appraisal: Include ☒ Exclude ☐ Seek further info ☐

Comments (Including reason for exclusion)

- Retrospective cohort study of 250 Brazilian paediatric SCA patients (125 male, 125 female) of average age 8.86±0.19 (5–16 yrs). Analysis of many parameters for specific BCL11A, HIMP-2A and HIMP-2B regions, HBBP1 and OR51B5/6 SNVs, including also ACS, hospitalization owing to pain and infection.

Critical Appraisal Checklist for Cohort Studies - © JBI, 2020. All rights reserved. JBI grants use of these tools for research purposes only. All other enquiries should be sent to jbisynthesis@adelaide.edu.au.

JBI CRITICAL APPRAISAL CHECKLIST FOR COHORT STUDIES

Reviewer: __Carsten W Lederer___________________ Date: _2024-06-03 ___________________________

Author: _Higgs et al. (DOI: 10.1056/NEJM198206173062402)__ Year: 1982_ Record Number: CW

	Yes	No	Unclear	Not applicable
1. Were the groups similar and recruited from the same population?	X			
2. Were the exposures measured similarly to assign people to both exposed and unexposed groups?	X			
3. Was the exposure measured in a valid and reliable way?	X			
4. Were confounding factors identified?	X			
5. Were strategies to deal with confounding factors stated?	X			
6. Were all groups/participants free of the outcome at the start of the study (or at the moment of exposure)?			X	
7. Were the outcomes measured in a valid and reliable way?	X			
8. Was the follow up time reported and sufficient to be long enough for outcomes to occur?	X			
9. Was follow up complete, and if not, were the reasons to loss to follow up described and explored?	X			
10. Were strategies to address incomplete follow up utilized?				X
11. Was appropriate statistical analysis used?			X	

Overall appraisal: Include [X] Exclude [ ] Seek further info [ ]

Comments (Including reason for exclusion)

- A UK cohort study based on 176 SCA patients, 88 with normal HBA1/HBA2 genotype, 44 heterozygous for a single α-globin gene deletion, 44 homozygous for a single α-globin gene deletion, matched across groups for age and sex. While p values for odds ratios were not provided, they can be calculated for several SCD-related phenomena, including ACS, based on the provided patient numbers.

Critical Appraisal Checklist for Cohort Studies - © JBI, 2020. All rights reserved. JBI grants use of these tools for research purposes only. All other enquiries should be sent to jbisynthesis@adelaide.edu.au.

JBI CRITICAL APPRAISAL CHECKLIST FOR COHORT STUDIES

Reviewer: __Carsten W Lederer____________________ Date: _2024-06-03 ___________________________

Author: Chang et al. _(DOI: 10.1046/j.1365-2141.1997.d01-2094.x)_Year: 1997 _Record Number: CX

	Yes	No	Unclear	Not applicable
1. Were the groups similar and recruited from the same population?	☒	☐	☐	☐
2. Were the exposures measured similarly to assign people to both exposed and unexposed groups?	☒	☐	☐	☐
3. Was the exposure measured in a valid and reliable way?	☒	☐	☐	☐
4. Were confounding factors identified?	☒	☐	☐	☐
5. Were strategies to deal with confounding factors stated?	☒	☐	☐	☐
6. Were all groups/participants free of the outcome at the start of the study (or at the moment of exposure)?	☐	☒	☐	☐
7. Were the outcomes measured in a valid and reliable way?	☒	☐	☐	☐
8. Was the follow up time reported and sufficient to be long enough for outcomes to occur?	☒	☐	☐	☐
9. Was follow up complete, and if not, were the reasons to loss to follow up described and explored?	☒	☐	☐	☐
10. Were strategies to address incomplete follow up utilized?	☐	☐	☐	☒
11. Was appropriate statistical analysis used?	☒	☐	☐	☐

Overall appraisal: Include ☐ Exclude ☒ Seek further info ☐

Comments (Including reason for exclusion)

- A cohort study of 112 SCA patients (64 male, 44 female) selected for homozygosity of haplotypes for what is termed the F-cell production (FCP) locus, also termed HBFQTL3 on Xp22.2. Meta-analyses with a Jamaican cohort are also included. **The study does not directly address any of the disease aspects of relevance for this review, but has been included in the text for influence of HBB alleles.**

Critical Appraisal Checklist for Cohort Studies - © JBI, 2020. All rights reserved. JBI grants use of these tools for research purposes only. All other enquiries should be sent to jbisynthesis@adelaide.edu.au.

JBI CRITICAL APPRAISAL CHECKLIST FOR COHORT STUDIES

Reviewer: Carsten W Lederer Date: 2024-06-03

Author: Kutlar et al. (DOI: 10.1081/hem-100104029) Year: 2001 Record Number: CY

	Yes	No	Unclear	Not applicable
1. Were the groups similar and recruited from the same population?	☒	☐	☐	☐
2. Were the exposures measured similarly to assign people to both exposed and unexposed groups?	☒	☐	☐	☐
3. Was the exposure measured in a valid and reliable way?	☒	☐	☐	☐
4. Were confounding factors identified?	☒	☐	☐	☐
5. Were strategies to deal with confounding factors stated?	☐	☐	☒	☐
6. Were all groups/participants free of the outcome at the start of the study (or at the moment of exposure)?	☐	☒	☐	☐
7. Were the outcomes measured in a valid and reliable way?	☒	☐	☐	☐
8. Was the follow up time reported and sufficient to be long enough for outcomes to occur?	☒	☐	☐	☐
9. Was follow up complete, and if not, were the reasons to loss to follow up described and explored?	☒	☐	☐	☐
10. Were strategies to address incomplete follow up utilized?	☐	☐	☐	☒
11. Was appropriate statistical analysis used?	☐	☒	☐	☐

Overall appraisal: Include ☒ Exclude ☐ Seek further info ☐

Comments (Including reason for exclusion)

- A cohort study of 107 SCA patients (41 male, 66 female), 45 with avascular necrosis (18 male, 27 female), 62 without avascular necrosis (23 male, 39 female), average ages and sex distribution are matched AVN vs non-AVN. While p values for odds ratios were not provided, they can be calculated for AVS based on the provided patient numbers for the MTHFR mutation and "α-thal" (assumed to be carrier status).

Critical Appraisal Checklist for Cohort Studies - © JBI, 2020. All rights reserved. JBI grants use of these tools for research purposes only. All other enquiries should be sent to jbisynthesis@adelaide.edu.au.

JBI CRITICAL APPRAISAL CHECKLIST FOR COHORT STUDIES

Reviewer: Carsten W Lederer Date: 2024-06-03

Author: Andrade et al. DOI faulty (PMID: 9723576) Year: 1998 Record Number: CZ

	Yes	No	Unclear	Not applicable
1. Were the groups similar and recruited from the same population?	☒	☐	☐	☐
2. Were the exposures measured similarly to assign people to both exposed and unexposed groups?	☒	☐	☐	☐
3. Was the exposure measured in a valid and reliable way?	☒	☐	☐	☐
4. Were confounding factors identified?	☒	☐	☐	☐
5. Were strategies to deal with confounding factors stated?	☒	☐	☐	☐
6. Were all groups/participants free of the outcome at the start of the study (or at the moment of exposure)?	☐	☒	☐	☐
7. Were the outcomes measured in a valid and reliable way?	☒	☐	☐	☐
8. Was the follow up time reported and sufficient to be long enough for outcomes to occur?	☒	☐	☐	☐
9. Was follow up complete, and if not, were the reasons to loss to follow up described and explored?	☒	☐	☐	☐
10. Were strategies to address incomplete follow up utilized?	☐	☐	☐	☒
11. Was appropriate statistical analysis used?	☐	☐	☒	☐

Overall appraisal: Include ☐ Exclude ☒ Seek further info ☐

Comments (Including reason for exclusion)

- A US cohort study of SCD patients (33 male, 40 female), 32.3 yrs (14-16) analysed the effect of MTHFR, Factor V Leiden and Prothromin mutations on different disease aspects, including avascular necrosis and priapism. **No significant associations were found, contributed to by a low n and a mixture of β-globin SCD genotypes.**

Critical Appraisal Checklist for Cohort Studies - © JBI, 2020. All rights reserved. JBI grants use of these tools for research purposes only. All other enquiries should be sent to jbisynthesis@adelaide.edu.au.

JBI CRITICAL APPRAISAL CHECKLIST FOR COHORT STUDIES

Reviewer: Carsten W Lederer Date: 2024-06-03

Author: Moreira Neto et al. (DOI: 10.1590/s0100-879x2006001000004) Year: 2006 Record Number: DA

	Yes	No	Unclear	Not applicable
1. Were the groups similar and recruited from the same population?	☐	☐	☒	☐
2. Were the exposures measured similarly to assign people to both exposed and unexposed groups?	☒	☐	☐	☐
3. Was the exposure measured in a valid and reliable way?	☒	☐	☐	☐
4. Were confounding factors identified?	☒	☐	☐	☐
5. Were strategies to deal with confounding factors stated?	☒	☐	☐	☐
6. Were all groups/participants free of the outcome at the start of the study (or at the moment of exposure)?	☐	☒	☐	☐
7. Were the outcomes measured in a valid and reliable way?	☒	☐	☐	☐
8. Was the follow up time reported and sufficient to be long enough for outcomes to occur?	☒	☐	☐	☐
9. Was follow up complete, and if not, were the reasons to loss to follow up described and explored?	☒	☐	☐	☐
10. Were strategies to address incomplete follow up utilized?	☐	☐	☐	☒
11. Was appropriate statistical analysis used?	☒	☐	☐	☐

Overall appraisal: Include ☒ Exclude ☐ Seek further info ☐

Comments (Including reason for exclusion)

- A cohort study of 58 SCD patients (29 SCA, 24 HbSC), with a considerable (though non-significant, p=0.059) age difference for SCA (28, 13-52) to HbSC (38.5, 17-22). Only combined phenotypes including AVN result in statistically significant findings for the MTHFR C677T mutation.

Critical Appraisal Checklist for Cohort Studies - © JBI, 2020. All rights reserved. JBI grants use of these tools for research purposes only. All other enquiries should be sent to jbisynthesis@adelaide.edu.au.

JBI CRITICAL APPRAISAL CHECKLIST FOR COHORT STUDIES

Reviewer: __Carsten W Lederer__________________ Date: _2024-06-03 ___________________________

Author: Zimmerman et al. [DOI: 10.1002/(sici)1096-8652(199812)59:4<267::aid-ajh1>3.0.co;2-w]
Year: _1998_ Record Number: DB

	Yes	No	Unclear	Not applicable
1. Were the groups similar and recruited from the same population?	☒	☐	☐	☐
2. Were the exposures measured similarly to assign people to both exposed and unexposed groups?	☒	☐	☐	☐
3. Was the exposure measured in a valid and reliable way?	☒	☐	☐	☐
4. Were confounding factors identified?	☐	☐	☒	☐
5. Were strategies to deal with confounding factors stated?	☐	☒	☐	☐
6. Were all groups/participants free of the outcome at the start of the study (or at the moment of exposure)?	☐	☒	☐	☐
7. Were the outcomes measured in a valid and reliable way?	☒	☐	☐	☐
8. Was the follow up time reported and sufficient to be long enough for outcomes to occur?	☒	☐	☐	☐
9. Was follow up complete, and if not, were the reasons to loss to follow up described and explored?	☒	☐	☐	☐
10. Were strategies to address incomplete follow up utilized?	☐	☐	☐	☒
11. Was appropriate statistical analysis used?	☐	☐	☒	☐

Overall appraisal: Include ☐ Exclude ☒ Seek further info ☐

Comments (Including reason for exclusion)

- A cohort study of mixed SCA, HbSC and HbSβ0 genotypes investigating the role of MTHFR C677T and C1565T on SCD-related disease complications, including AVN and stroke. **No significant associations were found, contributed to by a low n and a mixture of β-globin SCD genotypes**, and the provided numbers do not allow meta- or re-analysis.

Critical Appraisal Checklist for Cohort Studies - © JBI, 2020. All rights reserved. JBI grants use of these tools for research purposes only. All other enquiries should be sent to jbisynthesis@adelaide.edu.au.

JBI CRITICAL APPRAISAL CHECKLIST FOR CASE CONTROL STUDIES

Reviewer: Carsten W Lederer Date: 2024-06-03

Author: Castro et al. (DOI: 10.1111/j.1423-0410.2004.00536.x) Year: 2004 Record Number: DC

	Yes	No	Unclear	Not applicable
11. Were the groups comparable other than the presence of disease in cases or the absence of disease in controls?	☒	☐	☐	☐
12. Were cases and controls matched appropriately?	☒	☐	☐	☐
13. Were the same criteria used for identification of cases and controls?	☒	☐	☐	☐
14. Was exposure measured in a standard, valid and reliable way?	☒	☐	☐	☐
15. Was exposure measured in the same way for cases and controls?	☒	☐	☐	☐
16. Were confounding factors identified?	☒	☐	☐	☐
17. Were strategies to deal with confounding factors stated?	☒	☐	☐	☐
18. Were outcomes assessed in a standard, valid and reliable way for cases and controls?	☒	☐	☐	☐
19. Was the exposure period of interest long enough to be meaningful?	☒	☐	☐	☐
20. Was appropriate statistical analysis used?	☒	☐	☐	☐

Overall appraisal: Include ☒ Exclude ☐ Seek further info ☐

Comments (Including reason for exclusion)

- A Brazilian cohort study of 97 SCA patients (49 males, 48 females) of African descent, 27 yrs, 14-66 was divided for a case control study into patients with and without occlusive vascular complications. Association of variants of the human platelet antigen system (HPA) were investigated for association with VOE events (VOE+ n=34, VOE- n=63).

Critical Appraisal Checklist for Cohort Studies - © JBI, 2020. All rights reserved. JBI grants use of these tools for research purposes only. All other enquiries should be sent to jbisynthesis@adelaide.edu.au.

JBI CRITICAL APPRAISAL CHECKLIST FOR CASE CONTROL STUDIES

Reviewer: Carsten W Lederer Date: 2024-06-03

Author: Ulug et al. (DOI: 10.1007/s00277-008-0659-5) Year: 2009 Record Number: DD

	Yes	No	Unclear	Not applicable
1. Were the groups comparable other than the presence of disease in cases or the absence of disease in controls?	☒	☐	☐	☐
2. Were cases and controls matched appropriately?	☒	☐	☐	☐
3. Were the same criteria used for identification of cases and controls?	☒	☐	☐	☐
4. Was exposure measured in a standard, valid and reliable way?	☒	☐	☐	☐
5. Was exposure measured in the same way for cases and controls?	☒	☐	☐	☐
6. Were confounding factors identified?	☒	☐	☐	☐
7. Were strategies to deal with confounding factors stated?	☒	☐	☐	☐
8. Were outcomes assessed in a standard, valid and reliable way for cases and controls?	☒	☐	☐	☐
9. Was the exposure period of interest long enough to be meaningful?	☒	☐	☐	☐
10. Was appropriate statistical analysis used?	☒	☐	☐	☐

Overall appraisal: Include ☒ Exclude ☐ Seek further info ☐

Comments (Including reason for exclusion)

- A UK case-control study (AVN n=39, 38.82, 19–65; non-AVN n=205, 35.9, 15–84) with a mixture of SCA, HbSC, HbSβ-thalassemia genotypes investigating the association of several genes and SNVs with avascular necrosis.

Critical Appraisal Checklist for Cohort Studies - © JBI, 2020. All rights reserved. JBI grants use of these tools for research purposes only. All other enquiries should be sent to jbisynthesis@adelaide.edu.au.

JBI CRITICAL APPRAISAL CHECKLIST FOR CASE CONTROL STUDIES

Reviewer: Carsten W Lederer Date: 2024-06-03

Author: Adewoye et al. (DOI: 10.1086/506356) Year: 2006 Record Number: DE

	Yes	No	Unclear	Not applicable
1. Were the groups comparable other than the presence of disease in cases or the absence of disease in controls?	☒	☐	☐	☐
2. Were cases and controls matched appropriately?	☒	☐	☐	☐
3. Were the same criteria used for identification of cases and controls?	☒	☐	☐	☐
4. Was exposure measured in a standard, valid and reliable way?	☒	☐	☐	☐
5. Was exposure measured in the same way for cases and controls?	☒	☐	☐	☐
6. Were confounding factors identified?	☒	☐	☐	☐
7. Were strategies to deal with confounding factors stated?	☒	☐	☐	☐
8. Were outcomes assessed in a standard, valid and reliable way for cases and controls?	☒	☐	☐	☐
9. Was the exposure period of interest long enough to be meaningful?	☒	☐	☐	☐
10. Was appropriate statistical analysis used?	☒	☐	☐	☐

Overall appraisal: Include ☐ Exclude ☒ Seek further info ☐

Comments (Including reason for exclusion)

- A case-control study based on a subset of 1473 patients of the CSSCD US SCD cohort, testing only SCA patients (irrespective of α-thalassemia status), with cases those with hospitalization for bacteraemia over the average 5.2 years of study time (n=145; 18.3±14.9 yrs) and with controls patient who did not (n=1248; 21.2±13.5 yrs). However, **the study does not directly address any of the disease aspects of relevance for this review.**

Critical Appraisal Checklist for Cohort Studies - © JBI, 2020. All rights reserved. JBI grants use of these tools for research purposes only. All other enquiries should be sent to jbisynthesis@adelaide.edu.au.

JBI CRITICAL APPRAISAL CHECKLIST FOR COHORT STUDIES

Reviewer: Carsten W Lederer Date: 2024-06-03

Author: Sebastiani et al. (DOI: 10.1038/ng1533) Year: 2005 Record Number: DF

	Yes	No	Unclear	Not applicable
1. Were the groups similar and recruited from the same population?	☒	☐	☐	☐
2. Were the exposures measured similarly to assign people to both exposed and unexposed groups?	☒	☐	☐	☐
3. Was the exposure measured in a valid and reliable way?	☒	☐	☐	☐
4. Were confounding factors identified?	☒	☐	☐	☐
5. Were strategies to deal with confounding factors stated?	☒	☐	☐	☐
6. Were all groups/participants free of the outcome at the start of the study (or at the moment of exposure)?	☐	☒	☐	☐
7. Were the outcomes measured in a valid and reliable way?	☒	☐	☐	☐
8. Was the follow up time reported and sufficient to be long enough for outcomes to occur?	☒	☐	☐	☐
9. Was follow up complete, and if not, were the reasons to loss to follow up described and explored?	☒	☐	☐	☐
10. Were strategies to address incomplete follow up utilized?	☐	☐	☐	☒
11. Was appropriate statistical analysis used?	☒	☐	☐	☐

Overall appraisal: Include ☐ Exclude ☒ Seek further info ☐

Comments (Including reason for exclusion)

- A cohort study based on a subset of 1398 patients of the CSSCD US SCD cohort, testing only SCA patients (irrespective of α-thalassemia status) with at least one incidence of VOC in the time of registration, for the occurrence of stroke in relation to 108 SNPs in 39 genes. However, **the statistical analyses of this study were focussed on stroke alone and did not directly address any of the disease aspects of relevance for this review.**

Critical Appraisal Checklist for Cohort Studies - © JBI, 2020. All rights reserved. JBI grants use of these tools for research purposes only. All other enquiries should be sent to jbisynthesis@adelaide.edu.au.

JBI CRITICAL APPRAISAL CHECKLIST FOR COHORT STUDIES

Reviewer: Carsten W Lederer Date: 2024-06-04

Author: Chaar et al. DOI: n/a (PMID: 15710570) Year: 2005 Record Number: DG

	Yes	No	Unclear	Not applicable
1. Were the groups similar and recruited from the same population?	☒	☐	☐	☐
2. Were the exposures measured similarly to assign people to both exposed and unexposed groups?	☒	☐	☐	☐
3. Was the exposure measured in a valid and reliable way?	☒	☐	☐	☐
4. Were confounding factors identified?	☒	☐	☐	☐
5. Were strategies to deal with confounding factors stated?	☒	☐	☐	☐
6. Were all groups/participants free of the outcome at the start of the study (or at the moment of exposure)?	☐	☒	☐	☐
7. Were the outcomes measured in a valid and reliable way?	☒	☐	☐	☐
8. Was the follow up time reported and sufficient to be long enough for outcomes to occur?	☒	☐	☐	☐
9. Was follow up complete, and if not, were the reasons to loss to follow up described and explored?	☒	☐	☐	☐
10. Were strategies to address incomplete follow up utilized?	☐	☐	☐	☒
11. Was appropriate statistical analysis used?	☒	☐	☐	☐

Overall appraisal: Include ☐ Exclude ☒ Seek further info ☐

Comments (Including reason for exclusion)

- A cohort study for 324 SCA patients (171 paediatric, 153 adult) in Guadeloupe, with the goal of correlating UGT1A1 SNPs with unconjugated bilirubin levels and frequency of cholelithiasis. However**, the statistical analyses of this study were focussed on those disease aspects alone and did not directly address any of the disease aspects of relevance for this review. Included in the text for reference to cholelithiasis.**

Critical Appraisal Checklist for Cohort Studies - © JBI, 2020. All rights reserved. JBI grants use of these tools for research purposes only. All other enquiries should be sent to jbisynthesis@adelaide.edu.au.

JBI CRITICAL APPRAISAL CHECKLIST FOR CASE CONTROL STUDIES

Reviewer: Carsten W Lederer Date: 2024-06-04

Author: Nolan et al. (DOI: 10.1111/j.1365-2141.2006.06074.x) Year: 2006 Record Number: DH

	Yes	No	Unclear	Not applicable
1. Were the groups comparable other than the presence of disease in cases or the absence of disease in controls?	☒	☐	☐	☐
2. Were cases and controls matched appropriately?	☒	☐	☐	☐
3. Were the same criteria used for identification of cases and controls?	☒	☐	☐	☐
4. Was exposure measured in a standard, valid and reliable way?	☒	☐	☐	☐
5. Was exposure measured in the same way for cases and controls?	☒	☐	☐	☐
6. Were confounding factors identified?	☒	☐	☐	☐
7. Were strategies to deal with confounding factors stated?	☒	☐	☐	☐
8. Were outcomes assessed in a standard, valid and reliable way for cases and controls?	☒	☐	☐	☐
9. Was the exposure period of interest long enough to be meaningful?	☒	☐	☐	☐
10. Was appropriate statistical analysis used?	☒	☐	☐	☐

Overall appraisal: Include ☐ Exclude ☒ Seek further info ☐

Comments (Including reason for exclusion)

- A case-control study based on a subset of the CSSCD US SCD cohort restricted to patients of or over 20 yrs, testing patients with leg ulcers (n=387) against controls without incidence of leg ulcers (n=920). While priapism and ACS incidence were significantly elevated for leg ulcer cases against controls, **any of the 129 SNPs in 47 genes characterized for a subset of 243 cases vs 516 controls were only analysed for those two groups and not for any of the disease aspects of relevance for this review.**

Critical Appraisal Checklist for Cohort Studies - © JBI, 2020. All rights reserved. JBI grants use of these tools for research purposes only. All other enquiries should be sent to jbisynthesis@adelaide.edu.au.

JBI CRITICAL APPRAISAL CHECKLIST FOR COHORT STUDIES

Reviewer: __Carsten W Lederer____________________ Date: _2024-06-04 ___________________________

Author: Peters et al._ (DOI: 10.1210/rp.59.1.51)______________ Year: 2004_ Record Number: DI

	Yes	No	Unclear	Not applicable
1. Were the groups similar and recruited from the same population?	☐	☐	☐	☒
2. Were the exposures measured similarly to assign people to both exposed and unexposed groups?	☐	☐	☐	☒
3. Was the exposure measured in a valid and reliable way?	☐	☐	☐	☒
4. Were confounding factors identified?	☐	☐	☐	☒
5. Were strategies to deal with confounding factors stated?	☐	☐	☐	☒
6. Were all groups/participants free of the outcome at the start of the study (or at the moment of exposure)?	☐	☐	☐	☒
7. Were the outcomes measured in a valid and reliable way?	☐	☐	☐	☒
8. Was the follow up time reported and sufficient to be long enough for outcomes to occur?	☐	☐	☐	☒
9. Was follow up complete, and if not, were the reasons to loss to follow up described and explored?	☐	☐	☐	☒
10. Were strategies to address incomplete follow up utilized?	☐	☐	☐	☒
11. Was appropriate statistical analysis used?	☐	☐	☐	☒

Overall appraisal: Include ☐ Exclude ☒ Seek further info ☐

Comments (Including reason for exclusion)

- Inadvertently, this article is a review and should have been excluded from additional articles referenced by reviews in the original search. Moreover, none of the keywords of interest were identified in the article.

Critical Appraisal Checklist for Cohort Studies - © JBI, 2020. All rights reserved. JBI grants use of these tools for research purposes only. All other enquiries should be sent to jbisynthesis@adelaide.edu.au.

JBI CRITICAL APPRAISAL CHECKLIST FOR COHORT STUDIES

Reviewer: Carsten W Lederer Date: 2024-06-04

Author: Ashley-Koch et al. (DOI: 10.1182/blood-2007-02-074849) Year: 2008 Record Number: DJ

	Yes	No	Unclear	Not applicable
1. Were the groups similar and recruited from the same population?	☒	☐	☐	☐
2. Were the exposures measured similarly to assign people to both exposed and unexposed groups?	☒	☐	☐	☐
3. Was the exposure measured in a valid and reliable way?	☒	☐	☐	☐
4. Were confounding factors identified?	☒	☐	☐	☐
5. Were strategies to deal with confounding factors stated?	☒	☐	☐	☐
6. Were all groups/participants free of the outcome at the start of the study (or at the moment of exposure)?	☐	☒	☐	☐
7. Were the outcomes measured in a valid and reliable way?	☒	☐	☐	☐
8. Was the follow up time reported and sufficient to be long enough for outcomes to occur?	☒	☐	☐	☐
9. Was follow up complete, and if not, were the reasons to loss to follow up described and explored?	☒	☐	☐	☐
10. Were strategies to address incomplete follow up utilized?	☐	☐	☐	☒
11. Was appropriate statistical analysis used?	☒	☐	☐	☐

Overall appraisal: Include ☐ Exclude ☒ Seek further info ☐

Comments (Including reason for exclusion)

- A cohort of 518 adult US SCD patients of mixed SCA, HBSC, HbSβ-thalassemia genotype was shortlisted to 111 patients with SCA for statistical analyses of 297 SNPs in 49 genes involved in adhesion, signalling, transport or coagulation. However, those analyses were only performed in relation to pulmonary hypertensions, and **none of the parameters of interest for this review were statistically tested for association with the SNPs.**

Critical Appraisal Checklist for Cohort Studies - © JBI, 2020. All rights reserved. JBI grants use of these tools for research purposes only. All other enquiries should be sent to jbisynthesis@adelaide.edu.au.

JBI CRITICAL APPRAISAL CHECKLIST FOR COHORT STUDIES

Reviewer: __Carsten W Lederer__________________ Date: _2024-06-04 ___________________________

Author: Elliott et al. (DOI: 10.1111/j.1365-2141.2007.06560.x) __Year: 2007 _Record Number: DK

	Yes	No	Unclear	Not applicable
1. Were the groups similar and recruited from the same population?	☒	☐	☐	☐
2. Were the exposures measured similarly to assign people to both exposed and unexposed groups?	☒	☐	☐	☐
3. Was the exposure measured in a valid and reliable way?	☒	☐	☐	☐
4. Were confounding factors identified?	☐	☐	☒	☐
5. Were strategies to deal with confounding factors stated?	☒	☐	☐	☐
6. Were all groups/participants free of the outcome at the start of the study (or at the moment of exposure)?	☐	☒	☐	☐
7. Were the outcomes measured in a valid and reliable way?	☒	☐	☐	☐
8. Was the follow up time reported and sufficient to be long enough for outcomes to occur?	☒	☐	☐	☐
9. Was follow up complete, and if not, were the reasons to loss to follow up described and explored?	☒	☐	☐	☐
10. Were strategies to address incomplete follow up utilized?	☐	☐	☐	☒
11. Was appropriate statistical analysis used?	☐	☐	☒	☐

Overall appraisal: Include ☒ Exclude ☐ Seek further info ☐

Comments (Including reason for exclusion)

- A cohort of 190 male SCD patients of mixed SCA and HbSβ0-thalassemia genotype who indicated absence or presence of priapism, with no indication of age ranges. The study applied an FDR threshold of 0.1 to determine significance by multiple testing correction, which in particular for FDR-based adjustment is unusually lenient and a questionable choice.

Critical Appraisal Checklist for Cohort Studies - © JBI, 2020. All rights reserved. JBI grants use of these tools for research purposes only. All other enquiries should be sent to jbisynthesis@adelaide.edu.au.

JBI CRITICAL APPRAISAL CHECKLIST FOR CASE CONTROL STUDIES

Reviewer: __Carsten W Lederer__________________ Date: _2024-06-04 ___________________________

Author: Nolan et al. (DOI: 10.1111/j.1365-2141.2004.05295.x) __Year: 2005__Record Number: DL

	Yes	No	Unclear	Not applicable
1. Were the groups comparable other than the presence of disease in cases or the absence of disease in controls?	☒	☐	☐	☐
2. Were cases and controls matched appropriately?	☒	☐	☐	☐
3. Were the same criteria used for identification of cases and controls?	☒	☐	☐	☐
4. Was exposure measured in a standard, valid and reliable way?	☒	☐	☐	☐
5. Was exposure measured in the same way for cases and controls?	☒	☐	☐	☐
6. Were confounding factors identified?	☒	☐	☐	☐
7. Were strategies to deal with confounding factors stated?	☒	☐	☐	☐
8. Were outcomes assessed in a standard, valid and reliable way for cases and controls?	☒	☐	☐	☐
9. Was the exposure period of interest long enough to be meaningful?	☒	☐	☐	☐
10. Was appropriate statistical analysis used?	☒	☐	☐	☐

Overall appraisal: Include ☒ Exclude ☐ Seek further info ☐

Comments (Including reason for exclusion)

- A case-control study based on a subset of 677 males of the CSSCD US SCD cohort restricted to patients genotyped for the investigated SNPs, testing SCA patients with priapism (n=148) against controls without incidence of priapism (n=529).

Critical Appraisal Checklist for Cohort Studies - © JBI, 2020. All rights reserved. JBI grants use of these tools for research purposes only. All other enquiries should be sent to jbisynthesis@adelaide.edu.au.

JBI CRITICAL APPRAISAL CHECKLIST FOR CASE CONTROL STUDIES

Reviewer: Carsten W Lederer Date: 2024-06-04

Author: Nolan et al. (DOI: 10.1182/blood-2005-04-1594) Year: 2005 Record Number: DM

	Yes	No	Unclear	Not applicable
1. Were the groups comparable other than the presence of disease in cases or the absence of disease in controls?	☒	☐	☐	☐
2. Were cases and controls matched appropriately?	☒	☐	☐	☐
3. Were the same criteria used for identification of cases and controls?	☒	☐	☐	☐
4. Was exposure measured in a standard, valid and reliable way?	☒	☐	☐	☐
5. Was exposure measured in the same way for cases and controls?	☒	☐	☐	☐
6. Were confounding factors identified?	☒	☐	☐	☐
7. Were strategies to deal with confounding factors stated?	☒	☐	☐	☐
8. Were outcomes assessed in a standard, valid and reliable way for cases and controls?	☒	☐	☐	☐
9. Was the exposure period of interest long enough to be meaningful?	☒	☐	☐	☐
10. Was appropriate statistical analysis used?	☒	☐	☐	☐

Overall appraisal: Include ☐ Exclude ☒ Seek further info ☐

Comments (Including reason for exclusion)

- A case-control study based on a subset of 1252 males of the CSSCD US SCD cohort restricted to patients genotyped for the investigated SNPs, testing SCA patients with priapism (n=273) against controls without incidence of priapism (n=979). **However, the study only examined correlation of priapism with haemolysis phenotypes, without investigation of genetic variants.**

Critical Appraisal Checklist for Cohort Studies - © JBI, 2020. All rights reserved. JBI grants use of these tools for research purposes only. All other enquiries should be sent to jbisynthesis@adelaide.edu.au.

JBI CRITICAL APPRAISAL CHECKLIST FOR COHORT STUDIES

Reviewer: __Carsten W Lederer__________________ Date: _2024-06-04 __________________________

Author: Grasemann & Ratjen (DOI: 10.1111/j.1365-2141.2004.05001.x) Year: 2004 Record Number: DN

	Yes	No	Unclear	Not applicable
1. Were the groups similar and recruited from the same population?	☐	☐	☒	☐
2. Were the exposures measured similarly to assign people to both exposed and unexposed groups?	☐	☐	☒	☐
3. Was the exposure measured in a valid and reliable way?	☐	☐	☒	☐
4. Were confounding factors identified?	☐	☐	☒	☐
5. Were strategies to deal with confounding factors stated?	☐	☐	☒	☐
6. Were all groups/participants free of the outcome at the start of the study (or at the moment of exposure)?	☐	☐	☒	☐
7. Were the outcomes measured in a valid and reliable way?	☐	☐	☒	☐
8. Was the follow up time reported and sufficient to be long enough for outcomes to occur?	☐	☐	☒	☐
9. Was follow up complete, and if not, were the reasons to loss to follow up described and explored?	☐	☐	☒	☐
10. Were strategies to address incomplete follow up utilized?	☐	☐	☒	☐
11. Was appropriate statistical analysis used?	☐	☐	☒	☐

Overall appraisal: Include ☐ Exclude ☒ Seek further info ☐

Comments (Including reason for exclusion)

- This was a BHJ correspondence adding to the discussion of potentially sex-specific NOS1 and NOS3 involvement in SCD-related ACS. **No population statistics are given, and the authors merely point out their own data for cystic fibrosis**, which indicate a sex-specific contribution of a NOS3 variant to increased lung infections.

Critical Appraisal Checklist for Cohort Studies - © JBI, 2020. All rights reserved. JBI grants use of these tools for research purposes only. All other enquiries should be sent to jbisynthesis@adelaide.edu.au.

JBI CRITICAL APPRAISAL CHECKLIST FOR CASE CONTROL STUDIES

Reviewer: Carsten W Lederer Date: 2024-06-04

Author: Grasemann et al. (DOI: 10.1164/rccm.200211-1342OC) Year: 2003 Record Number: DO

	Yes	No	Unclear	Not applicable
1. Were the groups comparable other than the presence of disease in cases or the absence of disease in controls?	☒	☐	☐	☐
2. Were cases and controls matched appropriately?	☒	☐	☐	☐
3. Were the same criteria used for identification of cases and controls?	☒	☐	☐	☐
4. Was exposure measured in a standard, valid and reliable way?	☒	☐	☐	☐
5. Was exposure measured in the same way for cases and controls?	☒	☐	☐	☐
6. Were confounding factors identified?	☒	☐	☐	☐
7. Were strategies to deal with confounding factors stated?	☒	☐	☐	☐
8. Were outcomes assessed in a standard, valid and reliable way for cases and controls?	☒	☐	☐	☐
9. Was the exposure period of interest long enough to be meaningful?	☒	☐	☐	☐
10. Was appropriate statistical analysis used?	☒	☐	☐	☐

Overall appraisal: Include ☐ Exclude ☒ Seek further info ☐

Comments (Including reason for exclusion)

- This study, comparing smokers and non-smokers (total n=105) for airway nitric oxide concentrations in association with sex and NOS1 and NOS3 genes, makes **reference to SCD only in its discussion for related SCD data on sex-specific NOS1 action.**

Critical Appraisal Checklist for Cohort Studies - © JBI, 2020. All rights reserved. JBI grants use of these tools for research purposes only. All other enquiries should be sent to jbisynthesis@adelaide.edu.au.

JBI CRITICAL APPRAISAL CHECKLIST FOR COHORT STUDIES

Reviewer: __Carsten W Lederer__________________ Date: _2024-06-04 _________________________

Author: Sullivan et al. _(DOI: 10.1164/ajrccm.164.12.2012090) __Year: 2001 _Record Number: DP

	Yes	No	Unclear	Not applicable
1. Were the groups similar and recruited from the same population?	☐	☒	☐	☐
2. Were the exposures measured similarly to assign people to both exposed and unexposed groups?	☒	☐	☐	☐
3. Was the exposure measured in a valid and reliable way?	☒	☐	☐	☐
4. Were confounding factors identified?	☐	☐	☒	☐
5. Were strategies to deal with confounding factors stated?	☐	☒	☐	☐
6. Were all groups/participants free of the outcome at the start of the study (or at the moment of exposure)?	☐	☒	☐	☐
7. Were the outcomes measured in a valid and reliable way?	☒	☐	☐	☐
8. Was the follow up time reported and sufficient to be long enough for outcomes to occur?	☒	☐	☐	☐
9. Was follow up complete, and if not, were the reasons to loss to follow up described and explored?	☒	☐	☐	☐
10. Were strategies to address incomplete follow up utilized?	☐	☐	☐	☒
11. Was appropriate statistical analysis used?	☒	☐	☐	☐

Overall appraisal: Include ☐ Exclude ☒ Seek further info ☐

Comments (Including reason for exclusion)

- Small cohort study of SCD paediatric/juvenile patients (n=20, 6-18 yrs), with (n=13) and without (n=7) recent history of ACS, and healthy controls (n=6). **Sex distribution was different between groups, which for NOS1/3 is a concern. AAT repeat number as genetic analysis was correlated with exhaled nitric oxide levels as proxy for ACS analysis.**

Critical Appraisal Checklist for Cohort Studies - © JBI, 2020. All rights reserved. JBI grants use of these tools for research purposes only. All other enquiries should be sent to jbisynthesis@adelaide.edu.au.

JBI CRITICAL APPRAISAL CHECKLIST FOR COHORT STUDIES

Reviewer: Carsten W Lederer Date: 2024-06-04

Author: Zennadi et al. (DOI: 10.1182/blood-2004-01-0042) Year: 2004 Record Number: DQ

	Yes	No	Unclear	Not applicable
1. Were the groups similar and recruited from the same population?	☐	☐	☐	☒
2. Were the exposures measured similarly to assign people to both exposed and unexposed groups?	☐	☐	☐	☒
3. Was the exposure measured in a valid and reliable way?	☐	☐	☐	☒
4. Were confounding factors identified?	☐	☐	☐	☒
5. Were strategies to deal with confounding factors stated?	☐	☐	☐	☒
6. Were all groups/participants free of the outcome at the start of the study (or at the moment of exposure)?	☐	☐	☐	☒
7. Were the outcomes measured in a valid and reliable way?	☐	☐	☐	☒
8. Was the follow up time reported and sufficient to be long enough for outcomes to occur?	☐	☐	☐	☒
9. Was follow up complete, and if not, were the reasons to loss to follow up described and explored?	☐	☐	☐	☒
10. Were strategies to address incomplete follow up utilized?	☐	☐	☐	☒
11. Was appropriate statistical analysis used?	☐	☐	☐	☒

Overall appraisal: Include ☐ Exclude ☒ Seek further info ☐

Comments (Including reason for exclusion)

- This is a functional study not including patients or investigation of genetic variants, apart from analyses normal vs SCD. **Included in the text for areas of exploration.**

Critical Appraisal Checklist for Cohort Studies - © JBI, 2020. All rights reserved. JBI grants use of these tools for research purposes only. All other enquiries should be sent to jbisynthesis@adelaide.edu.au.

JBI CRITICAL APPRAISAL CHECKLIST FOR COHORT STUDIES

Reviewer: Carsten W Lederer Date: 2024-06-04

Author: Barrett-Connor et al. (DOI: 10.1016/0002-9343(68)90187-3) Year: 1968 Record Number: DR

	Yes	No	Unclear	Not applicable
1. Were the groups similar and recruited from the same population?	☐	☐	☐	☒
2. Were the exposures measured similarly to assign people to both exposed and unexposed groups?	☐	☐	☐	☒
3. Was the exposure measured in a valid and reliable way?	☐	☐	☐	☒
4. Were confounding factors identified?	☐	☐	☐	☒
5. Were strategies to deal with confounding factors stated?	☐	☐	☐	☒
6. Were all groups/participants free of the outcome at the start of the study (or at the moment of exposure)?	☐	☐	☐	☒
7. Were the outcomes measured in a valid and reliable way?	☐	☐	☐	☒
8. Was the follow up time reported and sufficient to be long enough for outcomes to occur?	☐	☐	☐	☒
9. Was follow up complete, and if not, were the reasons to loss to follow up described and explored?	☐	☐	☐	☒
10. Were strategies to address incomplete follow up utilized?	☐	☐	☐	☒
11. Was appropriate statistical analysis used?	☐	☐	☐	☒

Overall appraisal: Include ☐ Exclude ☒ Seek further info ☐

Comments (Including reason for exclusion)

- No molecular information is provided in this article. **Included in the text for reference to abdominal pain.**

Critical Appraisal Checklist for Cohort Studies - © JBI, 2020. All rights reserved. JBI grants use of these tools for research purposes only. All other enquiries should be sent to jbisynthesis@adelaide.edu.au.

JBI CRITICAL APPRAISAL CHECKLIST FOR COHORT STUDIES

Reviewer: __Carsten W Lederer___________________ Date: _2024-11-28 ___________________________

Author: Dovern et al. (DOI: 10.1016/j.jtct.2023.01.001)__________Year: 2023_ Record Number: DS

	Yes	No	Unclear	Not applicable
1. Were the groups similar and recruited from the same population?	☐	☐	☐	☒
2. Were the exposures measured similarly to assign people to both exposed and unexposed groups?	☒	☐	☐	☐
3. Was the exposure measured in a valid and reliable way?	☒	☐	☐	☐
4. Were confounding factors identified?	☐	☐	☐	☒
5. Were strategies to deal with confounding factors stated?	☐	☐	☐	☒
6. Were all groups/participants free of the outcome at the start of the study (or at the moment of exposure)?	☐	☒	☐	☐
7. Were the outcomes measured in a valid and reliable way?	☒	☐	☐	☐
8. Was the follow up time reported and sufficient to be long enough for outcomes to occur?	☒	☐	☐	☐
9. Was follow up complete, and if not, were the reasons to loss to follow up described and explored?	☒	☐	☐	☐
10. Were strategies to address incomplete follow up utilized?	☐	☐	☐	☒
11. Was appropriate statistical analysis used?	☒	☐	☐	☐

Overall appraisal: Include ☐ Exclude ☒ Seek further info ☐

Comments (Including reason for exclusion)

- This study was based on a small cohort (n=10) of 4 women and 6 men. No molecular information is provided in this article.

Critical Appraisal Checklist for Cohort Studies - © JBI, 2020. All rights reserved. JBI grants use of these tools for research purposes only. All other enquiries should be sent to jbisynthesis@adelaide.edu.au.

JBI CRITICAL APPRAISAL CHECKLIST FOR CASE REPORTS

Reviewer: Carsten W Lederer Date: 2024-11-28

Author: Meliti et al. (DOI: 10.7759/cureus.33594) Year: 2023 Record Number: DT

	Yes	No	Unclear	Not applicable
1. Were patient's demographic characteristics clearly described?	☒	☐	☐	☐
2. Was the patient's history clearly described and presented as a timeline?	☒	☐	☐	☐
3. Was the current clinical condition of the patient on presentation clearly described?	☒	☐	☐	☐
4. Were diagnostic tests or assessment methods and the results clearly described?	☒	☐	☐	☐
5. Was the intervention(s) or treatment procedure(s) clearly described?	☒	☐	☐	☐
6. Was the post-intervention clinical condition clearly described?	☒	☐	☐	☐
7. Were adverse events (harms) or unanticipated events identified and described?	☐	☐	☐	☒
8. Does the case report provide takeaway lessons?	☒	☐	☒	☐

Overall appraisal: Include ☐ Exclude ☒ Seek further info ☐

Comments (Including reason for exclusion)

- Case report of an 8-yr old boy with long-term undetected HbS/IVSI-6(T>C) and unusual clinical presentation. Takeaway lesson from this case is the need for vigilance and (timely) molecular follow-up for unusual patient phenotypes. **Included only in the section on β-genotypes.**

Critical Appraisal Checklist for Cohort Studies - © JBI, 2020. All rights reserved. JBI grants use of these tools for research purposes only. All other enquiries should be sent to jbisynthesis@adelaide.edu.au.

JBI CRITICAL APPRAISAL CHECKLIST FOR CASE REPORTS

Reviewer: __Carsten W Lederer____________________ Date: _2024-11-28 ___________________________

Author: Sepulveda et al. (DOI: 10.1080/08998280.2022.2155927) Year: 2023 Record Number: DU

	Yes	No	Unclear	Not applicable
1. Were patient's demographic characteristics clearly described?	☐	☒	☐	☐
2. Was the patient's history clearly described and presented as a timeline?	☒	☐	☐	☐
3. Was the current clinical condition of the patient on presentation clearly described?	☒	☐	☐	☐
4. Were diagnostic tests or assessment methods and the results clearly described?	☒	☐	☐	☐
5. Was the intervention(s) or treatment procedure(s) clearly described?	☒	☐	☐	☐
6. Was the post-intervention clinical condition clearly described?	☒	☐	☐	☐
7. Were adverse events (harms) or unanticipated events identified and described?	☒	☐	☐	☐
8. Does the case report provide takeaway lessons?	☐	☐	☒	☐

Overall appraisal: Include ☐ Exclude ☒ Seek further info ☐

Comments (Including reason for exclusion)

- A single 19-yr male with extreme symptoms of pain, cerebral fat embolism and with what was treated as multisystemic inflammatory syndrome in children. **Included only in the section on environmental factors.**

Critical Appraisal Checklist for Cohort Studies - © JBI, 2020. All rights reserved. JBI grants use of these tools for research purposes only. All other enquiries should be sent to jbisynthesis@adelaide.edu.au.

JBI CRITICAL APPRAISAL CHECKLIST FOR COHORT STUDIES

Reviewer: Carsten W Lederer Date: 2024-11-28

Author: Martin et al. (DOI: 10.1111/bjh.18867) Year: 2023 Record Number: DV

	Yes	No	Unclear	Not applicable
1. Were the groups similar and recruited from the same population?	☒	☐	☐	☐
2. Were the exposures measured similarly to assign people to both exposed and unexposed groups?	☒	☐	☐	☐
3. Was the exposure measured in a valid and reliable way?	☒	☐	☐	☐
4. Were confounding factors identified?	☒	☐	☐	☐
5. Were strategies to deal with confounding factors stated?	☒	☐	☐	☐
6. Were all groups/participants free of the outcome at the start of the study (or at the moment of exposure)?	☐	☒	☐	☐
7. Were the outcomes measured in a valid and reliable way?	☒	☐	☐	☐
8. Was the follow up time reported and sufficient to be long enough for outcomes to occur?	☒	☐	☐	☐
9. Was follow up complete, and if not, were the reasons to loss to follow up described and explored?	☒	☐	☐	☐
10. Were strategies to address incomplete follow up utilized?	☐	☐	☐	☒
11. Was appropriate statistical analysis used?	☒	☐	☒	☐

Overall appraisal: Include ☐ Exclude ☒ Seek further info ☐

Comments (Including reason for exclusion)

- A cohort study of 191 paediatric patients across the different waves of the COVID-19 pandemic from 3/2020 to 01/2022. No genetic modifiers were analysed, and the study is **only included in the text section on environmental factors.**

Critical Appraisal Checklist for Cohort Studies - © JBI, 2020. All rights reserved. JBI grants use of these tools for research purposes only. All other enquiries should be sent to jbisynthesis@adelaide.edu.au.

JBI CRITICAL APPRAISAL CHECKLIST FOR CASE REPORTS

Reviewer: Carsten W Lederer Date: 2024-12-01

Author: Orolu et al. (DOI: 10.4103/njcp.njcp_793_22) Year: 2023 Record Number: DW

	Yes	No	Unclear	Not applicable
1. Were patient's demographic characteristics clearly described?	☐	☒	☐	☐
2. Was the patient's history clearly described and presented as a timeline?	☒	☐	☐	☐
3. Was the current clinical condition of the patient on presentation clearly described?	☒	☐	☐	☐
4. Were diagnostic tests or assessment methods and the results clearly described?	☒	☐	☐	☐
5. Was the intervention(s) or treatment procedure(s) clearly described?	☒	☐	☐	☐
6. Was the post-intervention clinical condition clearly described?	☒	☐	☐	☐
7. Were adverse events (harms) or unanticipated events identified and described?	☒	☐	☐	☐
8. Does the case report provide takeaway lessons?	☐	☐	☒	☐

Overall appraisal: Include ☐ Exclude ☒ Seek further info ☐

Comments (Including reason for exclusion)

- A single 17-yr female with splenomegaly and a long history of abdominal swelling and upper abdominal pain was indicated for total splenectomy owing to extreme pain associated with a splenic pseudocyst. No genetic information or wider implications for the article were apparent. **Included in the text for reference to abdominal pain.**

Critical Appraisal Checklist for Cohort Studies - © JBI, 2020. All rights reserved. JBI grants use of these tools for research purposes only. All other enquiries should be sent to jbisynthesis@adelaide.edu.au.

JBI CRITICAL APPRAISAL CHECKLIST FOR COHORT STUDIES

Reviewer: Carsten W Lederer Date: 2024-12-01

Author: McClish et al. (DOI: 10.1097/j.pain.0000000000003011) Year: 2024 Record Number: DX

	Yes	No	Unclear	Not applicable
1. Were the groups similar and recruited from the same population?	☐	☐	☐	☒
2. Were the exposures measured similarly to assign people to both exposed and unexposed groups?	☒	☐	☐	☐
3. Was the exposure measured in a valid and reliable way?	☒	☐	☐	☐
4. Were confounding factors identified?	☒	☐	☐	☐
5. Were strategies to deal with confounding factors stated?	☒	☐	☐	☐
6. Were all groups/participants free of the outcome at the start of the study (or at the moment of exposure)?	☐	☒	☐	☐
7. Were the outcomes measured in a valid and reliable way?	☒	☐	☐	☐
8. Was the follow up time reported and sufficient to be long enough for outcomes to occur?	☒	☐	☐	☐
9. Was follow up complete, and if not, were the reasons to loss to follow up described and explored?	☒	☐	☐	☐
10. Were strategies to address incomplete follow up utilized?	☐	☐	☐	☒
11. Was appropriate statistical analysis used?	☐	☐	☒	☐

Overall appraisal: Include ☐ Exclude ☒ Seek further info ☐

Comments (Including reason for exclusion)

- A cohort study of 198 individuals ≥15 yrs investigated correlation of VOC intensity, frequency and duration with health-related quality of life. No analysis of genetic variants was undertaken and no other relevant conclusion were extracted for the present article.

Critical Appraisal Checklist for Cohort Studies - © JBI, 2020. All rights reserved. JBI grants use of these tools for research purposes only. All other enquiries should be sent to jbisynthesis@adelaide.edu.au.

JBI CRITICAL APPRAISAL CHECKLIST FOR CASE REPORTS

Reviewer: Carsten W Lederer Date: 2024-12-01

Author: Girish et al. (DOI: 10.12659/AJCR.941268) Year: 2024 Record Number: DY

	Yes	No	Unclear	Not applicable
1. Were patient's demographic characteristics clearly described?	☒	☐	☐	☐
2. Was the patient's history clearly described and presented as a timeline?	☒	☐	☐	☐
3. Was the current clinical condition of the patient on presentation clearly described?	☒	☐	☐	☐
4. Were diagnostic tests or assessment methods and the results clearly described?	☒	☐	☐	☐
5. Was the intervention(s) or treatment procedure(s) clearly described?	☒	☐	☐	☐
6. Was the post-intervention clinical condition clearly described?	☒	☐	☐	☐
7. Were adverse events (harms) or unanticipated events identified and described?	☒	☐	☐	☐
8. Does the case report provide takeaway lessons?	☐	☐	☒	☐

Overall appraisal: Include ☐ Exclude ☒ Seek further info ☐

Comments (Including reason for exclusion)

- A 21-yr female suffered a VOC, with intermittent asthma and hospitalization after her first use of an e-cigarette. No genetic information was provided and though smoking is a lifestyle choice likely to aggravate SCD symptoms and pain, no specific reference was included in the present article. **Included in the text for reference to likely cholelithiasis.**

Critical Appraisal Checklist for Cohort Studies - © JBI, 2020. All rights reserved. JBI grants use of these tools for research purposes only. All other enquiries should be sent to jbisynthesis@adelaide.edu.au.

JBI CRITICAL APPRAISAL CHECKLIST FOR SYSTEMATIC REVIEWS AND RESEARCH SYNTHESES

Reviewer: Carsten W Lederer Date: 2024-06-04

Author: Khamees et al. (DOI: 10.1186/s12967-021-02931-1) Year: 2021 Record Number: CC

	Yes	No	Unclear	Not applicable
1. Is the review question clearly and explicitly stated?	☒	☐	☐	☐
2. Were the inclusion criteria appropriate for the review question?	☒	☐	☐	☐
3. Was the search strategy appropriate?	☒	☐	☐	☐
4. Were the sources and resources used to search for studies adequate?	☒	☐	☐	☐
5. Were the criteria for appraising studies appropriate?	☐	☐	☒	☐
6. Was critical appraisal conducted by two or more reviewers independently?	☒	☐	☐	☐
7. Were there methods to minimize errors in data extraction?	☐	☐	☒	☐
8. Were the methods used to combine studies appropriate?	☐	☐	☒	☐
9. Was the likelihood of publication bias assessed?	☒	☐	☐	☐
10. Were recommendations for policy and/or practice supported by the reported data?	☒	☐	☐	☐
1. Were the specific directives for new research appropriate?	☒	☐	☐	☐

Overall appraisal: Include ☒ Exclude ☐ Seek further info ☐

Comments (Including reason for exclusion)

- A sound and comprehensive systematic review, strictly following JBI guidelines. With focus on HbSE and mostly individual case studies, this article was included only for reference to cholelithiasis.

Critical Appraisal Checklist for Cohort Studies - © JBI, 2020. All rights reserved. JBI grants use of these tools for research purposes only. All other enquiries should be sent to jbisynthesis@adelaide.edu.au.

JBI CRITICAL APPRAISAL CHECKLIST FOR SYSTEMATIC REVIEWS AND RESEARCH SYNTHESES

Reviewer: Carsten W Lederer Date: 2025-01-10

Author: Gehling et al. (DOI: 10.3389/fpain.2023.1223309) Year: 2023 Record Number: DZ

	Yes	No	Unclear	Not applicable
11. Is the review question clearly and explicitly stated?	☒	☐	☐	☐
12. Were the inclusion criteria appropriate for the review question?	☒	☐	☐	☐
13. Was the search strategy appropriate?	☒	☐	☐	☐
14. Were the sources and resources used to search for studies adequate?	☒	☐	☐	☐
15. Were the criteria for appraising studies appropriate?	☒	☐	☐	☐
16. Was critical appraisal conducted by two or more reviewers independently?	☒	☐	☐	☐
17. Were there methods to minimize errors in data extraction?	☐	☐	☒	☐
18. Were the methods used to combine studies appropriate?	☐	☐	☒	☐
19. Was the likelihood of publication bias assessed?	☐	☒	☐	☐
20. Were recommendations for policy and/or practice supported by the reported data?	☒	☐	☐	☐
2. Were the specific directives for new research appropriate?	☒	☐	☐	☐

Overall appraisal: Include ☒ Exclude ☐ Seek further info ☐

Comments (Including reason for exclusion)

- A systematic review, strictly following JBI guidelines. Inclusion of significant findings in Table 1 was supplemented with individual confirmation based on the primary articles, prompted by apparent carelessness in the review article. E.g., in its Table 1, references #28 and #35 and SNP lists for ADRB2 were mixed up, and the stated report of significant effects for acute pain for #28 was incorrect. Likewise, significance for AVPR1A in #35 was not in relation to pain phenotypes but in relation to SNP frequency in populations. Likewise, the reference given for PROZ is indexed wrongly (which should be #16, not #17). Likewise, the SNP listed in Table 1 for DRD3 with ID rs4680 would be in the COMT gene instead. Likewise, PNMT was consistently referred to as PMNT.

Critical Appraisal Checklist for Cohort Studies - © JBI, 2020. All rights reserved. JBI grants use of these tools for research purposes only. All other enquiries should be sent to jbisynthesis@adelaide.edu.au.

JBI CRITICAL APPRAISAL CHECKLIST FOR COHORT STUDIES

Reviewer: Carsten W Lederer Date: 2024-12-01

Author: Powell-Roach et al. (DOI: 10.1371/journal.pone.0224886) Year: 2019 Record Number: EA

	Yes	No	Unclear	Not applicable
12. Were the groups similar and recruited from the same population?	☐	☐	☐	☒
13. Were the exposures measured similarly to assign people to both exposed and unexposed groups?	☒	☐	☐	☐
14. Was the exposure measured in a valid and reliable way?	☒	☐	☐	☐
15. Were confounding factors identified?	☒	☐	☐	☐
16. Were strategies to deal with confounding factors stated?	☒	☐	☐	☐
17. Were all groups/participants free of the outcome at the start of the study (or at the moment of exposure)?	☐	☒	☐	☐
18. Were the outcomes measured in a valid and reliable way?	☒	☐	☐	☐
19. Was the follow up time reported and sufficient to be long enough for outcomes to occur?	☒	☐	☐	☐
20. Was follow up complete, and if not, were the reasons to loss to follow up described and explored?	☒	☐	☐	☐
21. Were strategies to address incomplete follow up utilized?	☐	☐	☐	☒
22. Was appropriate statistical analysis used?	☒	☐	☐	☐

Overall appraisal: Include ☒ Exclude ☐ Seek further info ☐

Comments (Including reason for exclusion)

- A cohort study of 107 American adults (35.2±12.0 yrs) with mixed SCD genotypes tested for rs10877969 genotype in conjunction with composite pain index and acute care utilization assessment.

Critical Appraisal Checklist for Cohort Studies - © JBI, 2020. All rights reserved. JBI grants use of these tools for research purposes only. All other enquiries should be sent to jbisynthesis@adelaide.edu.au.

JBI CRITICAL APPRAISAL CHECKLIST FOR COHORT STUDIES

Reviewer: Carsten W Lederer Date: 2024-12-01

Author: Jhun et al. (DOI: 10.1213/ANE.0000000000000382) Year: 2014 Record Number: EB

	Yes	No	Unclear	Not applicable
23. Were the groups similar and recruited from the same population?	☐	☐	☐	☒
24. Were the exposures measured similarly to assign people to both exposed and unexposed groups?	☒	☐	☐	☐
25. Was the exposure measured in a valid and reliable way?	☒	☐	☐	☐
26. Were confounding factors identified?	☒	☐	☐	☐
27. Were strategies to deal with confounding factors stated?	☒	☐	☐	☐
28. Were all groups/participants free of the outcome at the start of the study (or at the moment of exposure)?	☐	☒	☐	☐
29. Were the outcomes measured in a valid and reliable way?	☒	☐	☐	☐
30. Was the follow up time reported and sufficient to be long enough for outcomes to occur?	☒	☐	☐	☐
31. Was follow up complete, and if not, were the reasons to loss to follow up described and explored?	☐	☐	☐	☒
32. Were strategies to address incomplete follow up utilized?	☐	☐	☐	☒
33. Was appropriate statistical analysis used?	☒	☐	☐	☐

Overall appraisal: Include ☒ Exclude ☐ Seek further info ☐

Comments (Including reason for exclusion)

- An exploratory cohort study of 130 American adults (34.78±11.35 yrs) with mixed HbSS and HbSC SCD genotypes tested for DRD3 and COMT genotypes in conjunction with composite pain index and the number of acute care utilizations in the assessment period of 12 months post-pain assessment.

Critical Appraisal Checklist for Cohort Studies - © JBI, 2020. All rights reserved. JBI grants use of these tools for research purposes only. All other enquiries should be sent to jbisynthesis@adelaide.edu.au.

JBI CRITICAL APPRAISAL CHECKLIST FOR CASE CONTROL STUDIES

Reviewer: __Carsten W Lederer__________________ Date: _2024-12-01 ___________________________

Author: Belfer et al. (DOI: 10.1002/ajh.23613) __Year: 2013__Record Number: EC

	Yes	No	Unclear	Not applicable
11. Were the groups comparable other than the presence of disease in cases or the absence of disease in controls?	☒	☐	☐	☐
12. Were cases and controls matched appropriately?	☒	☐	☐	☐
13. Were the same criteria used for identification of cases and controls?	☒	☐	☐	☐
14. Was exposure measured in a standard, valid and reliable way?	☒	☐	☐	☐
15. Was exposure measured in the same way for cases and controls?	☒	☐	☐	☐
16. Were confounding factors identified?	☒	☐	☐	☐
17. Were strategies to deal with confounding factors stated?	☒	☐	☐	☐
18. Were outcomes assessed in a standard, valid and reliable way for cases and controls?	☒	☐	☐	☐
19. Was the exposure period of interest long enough to be meaningful?	☒	☐	☐	☐
20. Was appropriate statistical analysis used?	☒	☐	☐	☐

Overall appraisal: Include ☒ Exclude ☐ Seek further info ☐

Comments (Including reason for exclusion)

- A case control study of 228 SCD American adults with (32.4±10.0 yrs) and without (34.9±13.6) severe pain events tested for GCH1 rs8007267 genotypes, including comparison with subjects from the CSSCD cohort, in conjunction with composite pain index and the number of acute care utilizations in the assessment period of 12 months before pain assessment.

Critical Appraisal Checklist for Cohort Studies - © JBI, 2020. All rights reserved. JBI grants use of these tools for research purposes only. All other enquiries should be sent to jbisynthesis@adelaide.edu.au.

JBI CRITICAL APPRAISAL CHECKLIST FOR COHORT STUDIES

Reviewer: __Carsten W Lederer__________________ Date: _2024-12-01 ___________________________

Author: Jhun et al. (DOI: 10.2217/pgs-2017-0198) __Year: 2018 _Record Number: ED

	Yes	No	Unclear	Not applicable
1. Were the groups similar and recruited from the same population?	☐	☐	☐	☒
2. Were the exposures measured similarly to assign people to both exposed and unexposed groups?	☒	☐	☐	☐
3. Was the exposure measured in a valid and reliable way?	☒	☐	☐	☐
4. Were confounding factors identified?	☒	☐	☐	☐
5. Were strategies to deal with confounding factors stated?	☒	☐	☐	☐
6. Were all groups/participants free of the outcome at the start of the study (or at the moment of exposure)?	☐	☒	☐	☐
7. Were the outcomes measured in a valid and reliable way?	☒	☐	☐	☐
8. Was the follow up time reported and sufficient to be long enough for outcomes to occur?	☒	☐	☐	☐
9. Was follow up complete, and if not, were the reasons to loss to follow up described and explored?	☐	☐	☐	☒
10. Were strategies to address incomplete follow up utilized?	☐	☐	☐	☒
11. Was appropriate statistical analysis used?	☒	☐	☐	☐

Overall appraisal: Include ☒ Exclude ☐ Seek further info ☐

Comments (Including reason for exclusion)

- A study of 132 SCD American adults (34.2±11.8 yrs) with HbSS and HbSC genotypes, analysed for pain events within 12 months after assessment, in conjunction with for 8 TRPA1 and 3 TRPV1 variants.

Critical Appraisal Checklist for Cohort Studies - © JBI, 2020. All rights reserved. JBI grants use of these tools for research purposes only. All other enquiries should be sent to jbisynthesis@adelaide.edu.au.

JBI CRITICAL APPRAISAL CHECKLIST FOR COHORT STUDIES

Reviewer: Carsten W Lederer Date: 2024-12-01

Author: Jhun et al. (DOI: 10.2217/pgs-2018-0064) Year: 2018 Record Number: EE

	Yes	No	Unclear	Not applicable
12. Were the groups similar and recruited from the same population?	☐	☐	☐	☒
13. Were the exposures measured similarly to assign people to both exposed and unexposed groups?	☒	☐	☐	☐
14. Was the exposure measured in a valid and reliable way?	☒	☐	☐	☐
15. Were confounding factors identified?	☒	☐	☐	☐
16. Were strategies to deal with confounding factors stated?	☒	☐	☐	☐
17. Were all groups/participants free of the outcome at the start of the study (or at the moment of exposure)?	☐	☒	☐	☐
18. Were the outcomes measured in a valid and reliable way?	☒	☐	☐	☐
19. Was the follow up time reported and sufficient to be long enough for outcomes to occur?	☒	☐	☐	☐
20. Was follow up complete, and if not, were the reasons to loss to follow up described and explored?	☐	☐	☐	☒
21. Were strategies to address incomplete follow up utilized?	☐	☐	☐	☒
22. Was appropriate statistical analysis used?	☒	☐	☐	☐

Overall appraisal: Include ☒ Exclude ☐ Seek further info ☐

Comments (Including reason for exclusion)

- A study of 136 American adults (34.0±11.7 yrs) with mixed SCD genotypes, analysed for the number of acute care utilizations within 12 months after composite pain index assessment, in conjunction with analysis for 3 *NR3C1* variants.

Critical Appraisal Checklist for Cohort Studies - © JBI, 2020. All rights reserved. JBI grants use of these tools for research purposes only. All other enquiries should be sent to jbisynthesis@adelaide.edu.au.

JBI CRITICAL APPRAISAL CHECKLIST FOR COHORT STUDIES

Reviewer: __Carsten W Lederer___________________ Date: _2024-12-01 ___________________________

Author: Sadhu et al. (DOI: 10.2217/pgs-2019-0096) __Year: 2020 _Record Number: EF

	Yes	No	Unclear	Not applicable
23. Were the groups similar and recruited from the same population?	☐	☐	☐	☒
24. Were the exposures measured similarly to assign people to both exposed and unexposed groups?	☒	☐	☐	☐
25. Was the exposure measured in a valid and reliable way?	☒	☐	☐	☐
26. Were confounding factors identified?	☒	☐	☐	☐
27. Were strategies to deal with confounding factors stated?	☒	☐	☐	☐
28. Were all groups/participants free of the outcome at the start of the study (or at the moment of exposure)?	☐	☒	☐	☐
29. Were the outcomes measured in a valid and reliable way?	☒	☐	☐	☐
30. Was the follow up time reported and sufficient to be long enough for outcomes to occur?	☒	☐	☐	☐
31. Was follow up complete, and if not, were the reasons to loss to follow up described and explored?	☐	☐	☐	☒
32. Were strategies to address incomplete follow up utilized?	☐	☐	☐	☒
33. Was appropriate statistical analysis used?	☒	☐	☐	☐

Overall appraisal: Include ☒ Exclude ☐ Seek further info ☐

Comments (Including reason for exclusion)

- A study of 131 American adults (34.3±11.8 yrs) with mixed HbSS, HbSC and other genotypes, analysed for the number of acute care utilizations within 12 months after composite pain index assessment, in conjunction with analysis for 4 *PNMT* variants.

Critical Appraisal Checklist for Cohort Studies - © JBI, 2020. All rights reserved. JBI grants use of these tools for research purposes only. All other enquiries should be sent to jbisynthesis@adelaide.edu.au.

JBI CRITICAL APPRAISAL CHECKLIST FOR COHORT STUDIES

Reviewer: Carsten W Lederer Date: 2024-12-01

Author: Sadhu et al. (DOI: 10.1016/j.exphem.2018.07.004) Year: 2020 Record Number: EG

	Yes	No	Unclear	Not applicable
34. Were the groups similar and recruited from the same population?	☐	☐	☐	☒
35. Were the exposures measured similarly to assign people to both exposed and unexposed groups?	☒	☐	☐	☐
36. Was the exposure measured in a valid and reliable way?	☒	☐	☐	☐
37. Were confounding factors identified?	☒	☐	☐	☐
38. Were strategies to deal with confounding factors stated?	☒	☐	☐	☐
39. Were all groups/participants free of the outcome at the start of the study (or at the moment of exposure)?	☐	☒	☐	☐
40. Were the outcomes measured in a valid and reliable way?	☒	☐	☐	☐
41. Was the follow up time reported and sufficient to be long enough for outcomes to occur?	☒	☐	☐	☐
42. Was follow up complete, and if not, were the reasons to loss to follow up described and explored?	☐	☐	☐	☒
43. Were strategies to address incomplete follow up utilized?	☐	☐	☐	☒
44. Was appropriate statistical analysis used?	☒	☐	☐	☐

Overall appraisal: Include ☒ Exclude ☐ Seek further info ☐

Comments (Including reason for exclusion)

- A study of 131 American adults (34.3±11.8 yrs) with mixed HbSS, HbSC, HbSβ$^+$ and HbSβ^0 genotypes, analysed for the number of acute care utilizations within 12 months after composite pain index assessment, in conjunction with analysis for 5 *GCH1* variants.

Critical Appraisal Checklist for Cohort Studies - © JBI, 2020. All rights reserved. JBI grants use of these tools for research purposes only. All other enquiries should be sent to jbisynthesis@adelaide.edu.au.
